# Transcriptome of the synganglion in the tick *Ixodes ricinus* and evolution of the cys-loop ligand-gated ion channel family in ticks

**DOI:** 10.1186/s12864-022-08669-4

**Published:** 2022-06-23

**Authors:** Claude Rispe, Caroline Hervet, Nathalie de la Cotte, Romain Daveu, Karine Labadie, Benjamin Noel, Jean-Marc Aury, Steeve Thany, Emiliane Taillebois, Alison Cartereau, Anaïs Le Mauff, Claude L. Charvet, Clément Auger, Elise Courtot, Cédric Neveu, Olivier Plantard

**Affiliations:** 1 INRAE, Oniris, BIOEPAR, Nantes, France; 2 Department of Biology and Biotechnology “L. Spallanzani”, University of Pavia, Pavia, Italy; 3 Génomique Métabolique, Genoscope, Institut de biologie François Jacob, CEA, CNRS, Université d’Evry, Université Paris-Saclay, Evry, France; 4 Université d’Orléans, LBLGC USC INRAE 1328, 1 rue de Chartres, 45067 Orléans, France; 5 INRAE, ISP, 37380 Nouzilly, France

**Keywords:** Cys-loop receptors, Tick, Synganglion, Differential expression, Duplication

## Abstract

**Background:**

Ticks represent a major health issue for humans and domesticated animals. Exploring the expression landscape of the tick’s central nervous system (CNS), known as the synganglion, would be an important step in understanding tick physiology and in managing tick-borne diseases, but studies on that topic are still relatively scarce. Neuron-specific genes like the cys-loop ligand-gated ion channels (cys-loop LGICs, or cysLGICs) are important pharmacological targets of acaricides. To date their sequence have not been well catalogued for ticks, and their phylogeny has not been fully studied.

**Results:**

We carried out the sequencing of transcriptomes of the *I. ricinus* synganglion, for adult ticks in different conditions (unfed males, unfed females, and partially-fed females). The de novo assembly of these transcriptomes allowed us to obtain a large collection of cys-loop LGICs sequences. A reference meta-transcriptome based on synganglion and whole body transcriptomes was then produced, showing high completeness and allowing differential expression analyses between synganglion and whole body. Many of the genes upregulated in the synganglion were associated with neurotransmission and/or localized in neurons or the synaptic membrane. As the first step of a functional study of cysLGICs, we cloned the predicted sequence of the resistance to dieldrin (RDL) subunit homolog, and functionally reconstituted the first GABA-gated receptor of *Ixodes ricinus*. A phylogenetic study was performed for the nicotinic acetylcholine receptors (nAChRs) and other cys-loop LGICs respectively, revealing tick-specific expansions of some types of receptors (especially for Histamine-like subunits and GluCls).

**Conclusions:**

We established a large catalogue of genes preferentially expressed in the tick CNS, including the cysLGICs. We discovered tick-specific gene family expansion of some types of cysLGIC receptors, and a case of intragenic duplication, suggesting a complex pattern of gene expression among different copies or different alternative transcripts of tick neuro-receptors.

**Supplementary Information:**

The online version contains supplementary material available at 10.1186/s12864-022-08669-4.

## Background

Like the vertebrate brain, the central nervous system (CNS) of arthropods performs essential functions, such as the processing of stimuli, the control of movement and the regulation of many physiological processes. Whether one compares the brain of vertebrates and the CNS of arthropods, or compares the CNS of different arthropod groups, important similarities but also differences are evident, and can be related to the specific biology and evolution of each group [1]. In ticks, a group of haematophagous arthropods belonging to the Chelicerata, the synganglion constitutes the tick’s central nervous system, representing the fused ancestral brain and ventral nerve chord. It consists of a highly consolidated neuronal mass (cortex) surrounding innumerable axons and dendrites (neuropile) [2]. As ticks are potential transmitters of many pathogens and parasites, a better knowledge of the function of this essential organ could help in understanding interactions between ticks and tick-borne-pathogens [3], but also provide targets for chemical acaricides [4]. Indeed, to-date, most acaricides have targeted neural functions, but as with many insect pests, the continued use of these molecules has generated resistance, prompting the need to find new methods or targets [5].

Knowledge of the neural biology of ticks is scarce compared to other invertebrates [2], particularly with respect to gene expression changes in the synganglion during blood feeding or after mating [6]. However progress has been made, notably through the sequencing of synganglion transcriptomes of several tick species. For example, a study of expressed-sequence tags of the *Rhipicephalus sanguineus* synganglion has identified different neural receptors [7]. Two companion studies of this study reported the sequencing of synganglion transcriptomes of the American dog tick, *Dermacentor variabilis* [8, 9]. A synganglion transcriptome was also sequenced and annotated for *Ixodes scapularis* [6]. The above studies have identified several genes associated with the neural system and nerve impulse transmission, for example neuropeptides and proteins associated with neurotransmission. The synganglion transcriptome of the cattle tick, *Rhipicephalus microplus*, was investigated with a focus on predicting genes in the G protein-coupled receptor family [10], representing potential targets for new acaricides [11]. But in each of the above studies, it was impossible to assess the extent to which these gene collections were synganglion specific, as expression levels were only measured for synganglion samples, and were not compared to other tissues or the whole body. Recently, however, a study of the synganglion transcriptome in the tick *Rhipicephalus microplus* described tick neuropeptide sequences and demonstrated that they are strongly over-expressed in the synganglion compared to other tissues [12]. Finally, the *Ixodes scapularis* ISE6 cell line was shown to have neuronal characteristics, which provided a base for investigating neuronal interactions between ticks and pathogens [3]. In addition, Ligand-gated cys-loop ion channels (cys-loop LGICs) are key receptors mediating neurotransmission both in vertebrates and invertebrates. Ligand-gated ion channels are major pharmacological targets of pesticides or acaricides such as macrocyclic lactones, phenylpyrazoles and neonicotinoids [5]. Yet, their knowledge for ticks is partial in several aspects, including sequence reconstruction and annotation, phylogeny, or function, even though complete genomes of ticks have been sequenced for *Ixodes scapularis* [4] (32 sequences of cysLGICs were found in this study) or five other tick species [13] (no specific annotation of cysLGICs reported in this study). The cys-loop LGICs form a superfamily of subunits that share a similar membrane topology. Indeed, they comprise one large amino terminal ligand-binding domain and four transmembrane domains, the second of these domains determining the ionic pore. The ionic pore consists of a multimeric assembly of five subunits, which can be identical or different. Subgroups within LGICs are determined by the activating neurotransmitter molecules - acetylcholine, *γ*-Aminobutyric acid (GABA), glycine, glutamate, or histamine - able to bind and trigger the opening of the channel gate. In Chelicerata, a first extensive reconstruction and in-depth study of the cys-loop LGICs was carried out for the two-spotted spider mite *Tetranychus urticae* (Acari: Acariformes), a phytophagous parasite, on the basis of its genome sequence [14]. On the basis of its transcriptomes, a similar reconstruction and phylogenetic study was carried out for the spider *Cupiennius salei* [15]. Similar analysis is lacking for ticks, despite the important implications of this knowledge for a better understanding of tick neurobiology and for providing potential targets for their control. The tick *Ixodes ricinus* is a widespread species in Europe, where it represents an increasing human health concern, being the main potential transmitter of the Lyme disease agent and of other microbes or parasites [16]. No transcriptome of the synganglion has been sequenced and no annotation of the cys-loop LGICs has been yet performed for this species, to our knowledge.

To study the interactions between ion channels and pesticides, i.e. between the nicotinic receptors of acetylcholine (nAChRs) and neonicotinoids, and between GABA receptors and other molecules, and to evaluate possible adverse effects on non-target species, systems of ex-vivo expression constitute an approach of choice. However the heterologous expression of some ion channels such as cholinergic receptors remains challenging. This has been succesful in some arthropods using the alpha subunits of the target species combined with vertebrate beta subunits (making hybrid receptors), whereas a first non-hybrid arthropod receptor using alpha and beta units from the target species, was obtained for the salmon louse *Lepeophtheirus salmonis* [17]*.* For arachnids, the first functional characterization of an alpha-type receptor was done for the brown dog tick *Rhipicephalus sanguineus*, but in vitro expression patterns were unreliable [18]. In the tick *I. ricinus*, the microtransplatation of synganglion membranes in oocytes of *Xenopus levis* allowed to express nicotinic acetylcholine receptors and to evaluate their sensitivity to insecticides [19], but insofar no cloning of alpha-type receptor has been reported for ticks. A GABA receptor was first identified in *Drosophila melanogaster*, the *Rdl* gene [20], whereas *Rdl* homologs were cloned in the ticks *Dermacentor variabilis* [21] and *Rhipicephalus microplus* [22] respectively.

In the present study, our principal aims were i) to characterize genes that are specifically expressed (or up-regulated) in the synganglion of the tick *Ixodes ricinus* and ii) to carefully reconstruct the sequences of the *Ixodes ricinus* cys-loop LGICs, and to study their phylogeny including homologs in Arachnida and other arthropods.

## Results

### Detection of potential contaminants in the synganglion libraries

A high percentage of the reads were successfully assigned by Kraken 2 (~ 65% in the eight synganglion libraries, Table S1). The large majority of assignations were to hard ticks (Ixodidae). A probable contamination of a library (AA library, unfed ticks) by filarial nematods was noted, with 0.26% of the reads assigned to Filaroidea. A more detailed analysis of the contigs (searching homologs to the *co1* gene) suggest that the species was *Cercopithifilaria rugosicauda*, a nematode of the roe deer (*Capreolus capreolus*) transmitted in Europe by *Ixodes ricinus* [23]. It is unclear if some nematodes might have been present inside the dissected synganglia, or if nematode tissues present in other parts of the tick body contaminated our samples during dissection. We also note a potential contamination by Apicomplexa transmitted by ticks (*Babesia* sp.). For Bacteria, there were very low levels of contamination assigned to Spirochaetes (a group which includes Lyme disease agent, *Borrelliela* sp.) and to Rickettsiales, a group including both pathogens transmited by ticks (e.g. *Anaplasma* sp.) and a potential symbiont of *Ixodes ricinus*, *Candidatus* Midichloria mitochondrii [24]. Finally, one library contained viruses associated with ticks: this was analysed in a previous study where we obtained a complete genome of an iflavirus [25].

### Transcriptome assembly and reference transcriptome

After sequencing a total of 69.7 Gb, the reads of libraries corresponding to synganglions of ticks in “fed” and “unfed” conditions were assembled separately. Both assemblies were combined with two more assemblies recently obtained for whole ticks transcriptomes, to obtain a reference meta-transcriptome. Detailed assembly statistics for the meta-transcriptome and for its four components are given in Table 1. Numbers of contigs were > 600 K for the synganglion assemblies. The percentage of predicted complete BUSCO genes were relatively even among these four assemblies, ranging between 90.9 and 92.4%. The meta-transcriptome contained 70,125 contigs, a sharp decrease compared to the synganglion transcriptomes, which is explained by the filtering steps of Drap and the selection of contigs with a minimum read support, here 1 fpkm (Fragments Per Kilobase Million). The BUSCO completeness of the reference transcriptome (96.6%) was higher than any of the component assemblies, and the number of fragmented genes was very low (only 8).

**Table 1:**
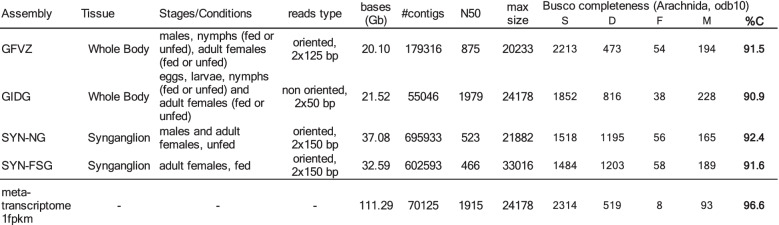
Statistics on transcriptome assemblies for *I. ricinus*, including two previously published datasets (transcriptomes of tick whole bodies), two transcriptome assemblies obtained for the tick synganglion (this study), and the reference meta-transcriptome obtained with Drap. Assembly short name (TSA accession for whole body transcriptomes), Tissue, stages, Read type (length and strandedness), Total sequenced bases, Number of contigs, N50 metric of each assembly, Maximum contig size, Busco completeness metrics, using the Arachnida odb10 data set (S, single and complete, D, duplicated and complete, F, fragmented, M, missing, %C, percentage of complete genes)

### Clustering of libraries based on read counts

**Fig. 1:**
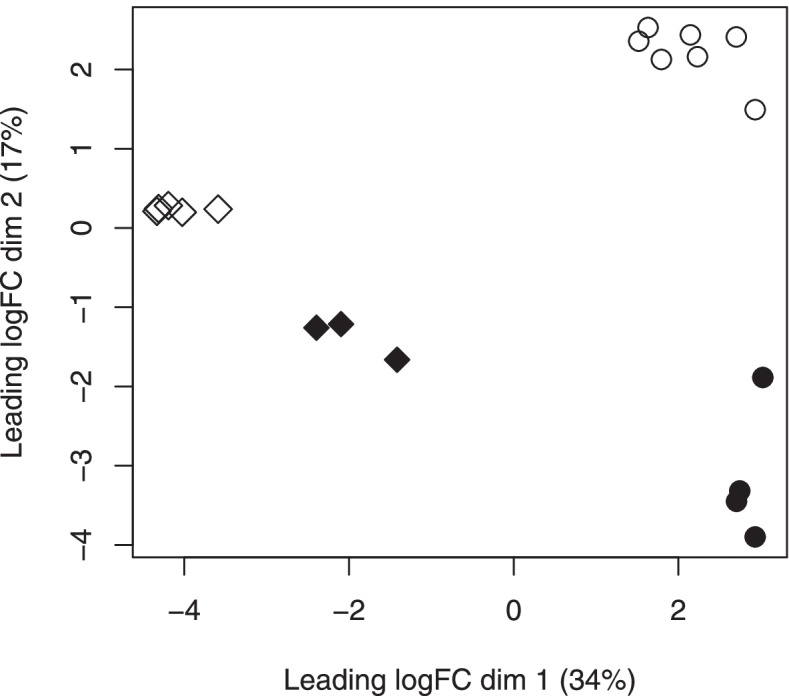
MDS plot showing the major sources of variation in expression counts. Plot shown for dimensions 1 and 2, the two axes with the highest weight. Synganglion libraries were produced in this study, whole body libraries were from a previous study (as described in Table 4). Synganglion of unfed ticks (open diamonds), synganglion of fed ticks (filled diamonds), whole body of unfed ticks (open circles), whole body of fed ticks (filled circles)

We observed a strong clustering of libraries that corresponded to the same combination of tissue and feeding status, suggesting that other factors (stage, sex) influenced comparatively less the levels of expression (MDS plot, Fig. 1). Indeed, the first axis of variation (axis 1, weight of 34%) clearly separated synganglion and whole body samples, whereas the second factor (axis 2, weight of 17%) separated the unfed and the partially fed conditions, respectively. Of note, expression levels were much less influenced by the feeding status in synganglion libraries, compared to whole body libraries. Positions of the “fed-synganglion” libraries in the MDS plot also indicate that these samples were less differentiated from whole-body libraries than the “unfed-synganglion” libraries (see Discussion). Given our focus on specific patterns of expression of the synganglion, we decided to make a differential expression analysis in two steps: first, we compared all synganglion libraries on one side versus all whole body libraries on the other side. Second, for the synganglion libraries only, we made a comparison betwen the “fed” and “unfed” samples, to evaluate the effect of the feeding status on expression levels in this tissue.

### Differential expression analysis, comparison synganglion vs whole body

**Fig. 2:**
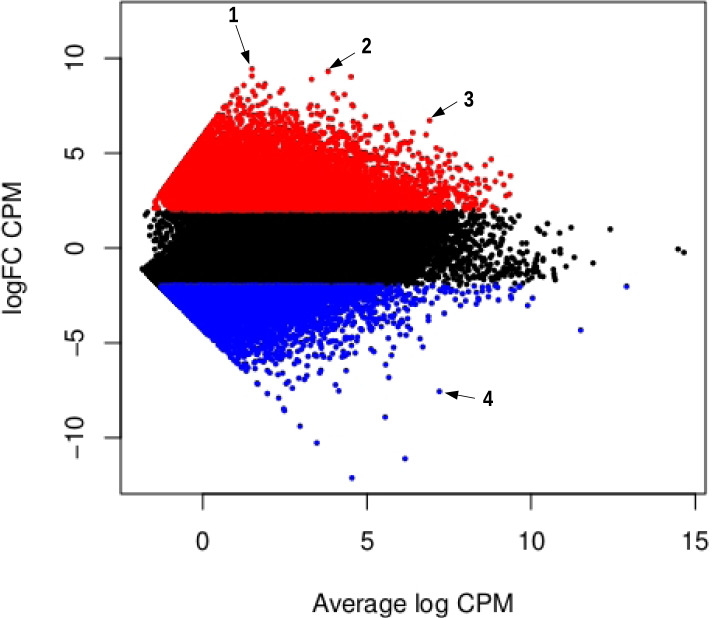
MD plot allowing to visualize expression differences between synganglion and whole body, for all genes. On the y-axis, log-fold change (FC) between the expression level in the synganglion and in the whole body, measured in counts per million (cpm). On the x-axis: log count per million of reads averaged across all libraries, indicating overall level of expression Dots in red correspond to genes that are significantly up-regulated in the synganglion (logfold change > 2 and *P*-adjusted value < 0.05); dots in blue correspond to genes that are significantly down-regulated in the synganglion (logfold change < 2 and *P*-adjusted value < 0.05); dots in gray are genes with an expression level not signicantly different among tissues. Three outliers with among the highest values of logfold-change are distinguished (blastx description betwen parentheses): 1 (Putative apoptosis-promoting rna-binding protein tia-1/tiar rrm superfamily), 2 (Putative beta-amyloid-like protein), 3 (Microtubule-associated protein tau), 4 (Cathepsin B)

**Fig. 3:**
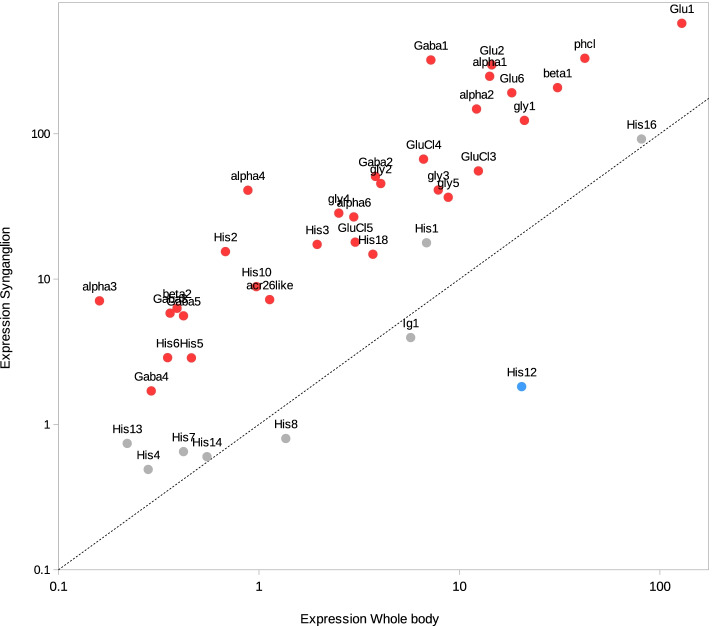
Comparison of expression counts for synganglion and whole body, for the cys-loop LCICs. Expression was measured in counts per million reads (cpm) with RSEM. Expression counts were averaged for all synganglion libraries (y-axis) and for all whole body libraries (x-axis). Colors of dots indicate if genes were found to be significantly up-regulated in the synganglion (red), down-regulated in the synganglion (blue) or with no sig. Difference (grey). The dotted-line corresponds to y = x. For both axes, a logarithmic scale was used. Corresponding accessions are listed in Table S6

A total of 8483 genes were up-regulated in the synganglion (Syn+ genes) while 5040 genes were down-regulated (Syn- genes), the remaining 22,592 genes showing no significant difference between the synganglion and whole body samples. Therefore ~ 37% of genes were found to be differently expressed among tissues. Many GO term associated with up-regulated genes for the Biogical Process (BP) and Molecular Function (MF) categories were consistent with neuronal functions (Table 2). This is the case of the terms GO:0098632 (cell-cell adhesion mediator activity), GO:1904315 (transmitter-gated ion channel activity involved in regulation of postsynaptic membrane potential, a GO term associated with cys-loop LGICs). As for the localization of expression (Cellular component, CC), many of the enriched terms were consistent with post-synaptic membranes or neurons. We also note the importance of terms associated with a large family of membrane-associated proteins, the G protein-coupled receptors. A mean-difference plot (MD plot) also allowed to visualize genes with a significantly change of expression level in both tissues (Fig. 2). Among genes with the highest over-expression in the synganglion, we note the cases of three genes of interest (Putative apoptosis-promoting rna-binding protein tia-1/tiar rrm superfamily, Putative beta-amyloid-like protein, Microtubule-associated protein tau). Most of the cys-loop LGICs were characterized by a strong over-expression in the synganglion (Fig. 3): this included the GABA-1 (*Rdl*) receptor, and the eight nAChRs. We note that three of the nAChRs (beta1, alpha1 and alpha2) had very high and roughly equal levels of expression in the synganglion, suggesting a possible co-expression and that these three genes could form heteromeric receptors. By contrast, the levels of expression of some of the histamine receptors were not significantly different between the synganglion and whole body samples respectively, as was the case of Ig1, the homolog of CG7589, CG6927, and CG11340 (forming the clade Insect group 1, in the house-fly).

**Table 2:**
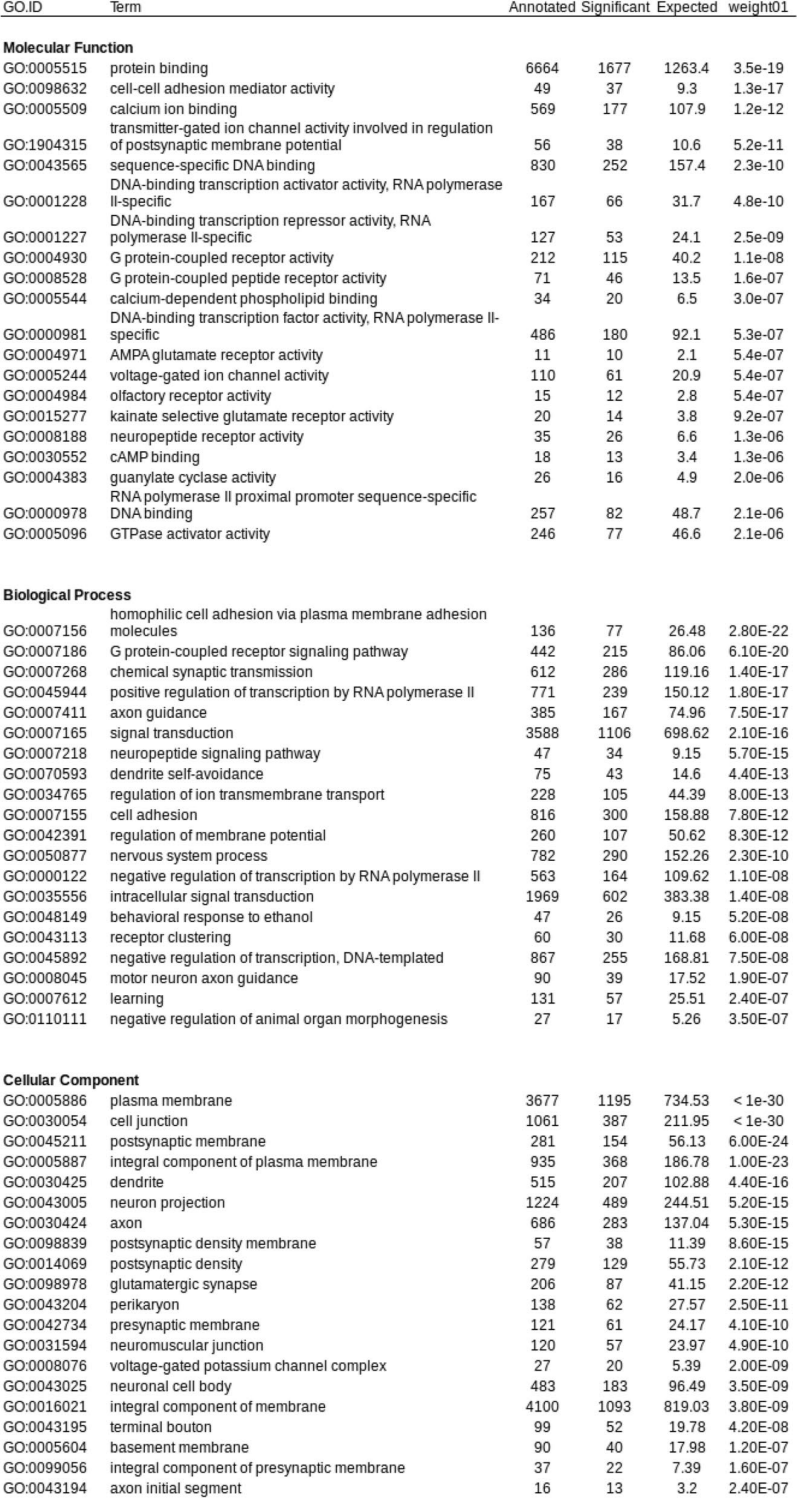
Top 20 enriched Gene Ontology terms among genes significantly up-regulated in the synganglion, compared to the whole body. Columns, GO IDs and terms for Molecular Function, Biological Process and Cellular localization; Annotated: number of transcripts with each GO; Significant: number of genes annotated with this term in the enriched category. Expected: expected number of genes. Weight01: level of significance of the enrichment

For the MF category, GO terms enriched in genes down-regulated in the synganglion (*n* = 5040) included several linked terms (associated to the same genes): the first three categories (Iron ion biding, heme binding, aromatase activity) were indeed often found for genes belonging to the cytochrome P450 family (Table S2).

### Comparison fed vs unfed ticks

A total of 2243 genes were up-regulated in the fed condition (Fed+ genes), whereas 2305 genes were down-regulated (Fed- genes) - for the remaining 35,667 genes, the expressions levels did not differ significantly between the fed and unfed samples. This amounted to ~ 11% of genes differently expressed among feeding conditions. The functional profiles of Fed+ genes showed notably an enrichment in genes associated with the metabolism of chitin and with the cuticle (Table S3), whereas Fed- genes were enriched in a variety of GO terms (Table S4), several of them being also enriched in the Syn+ category defined in the Synganglion vs Whole body comparison (e.g. terms associated with the synapse and with the plasma membrane). Among Syn+ genes, 1362 were Fed- whereas only 79 were Fed+.

### Phylogeny of AChRs from ticks and other Arachnida

**Fig. 4:**
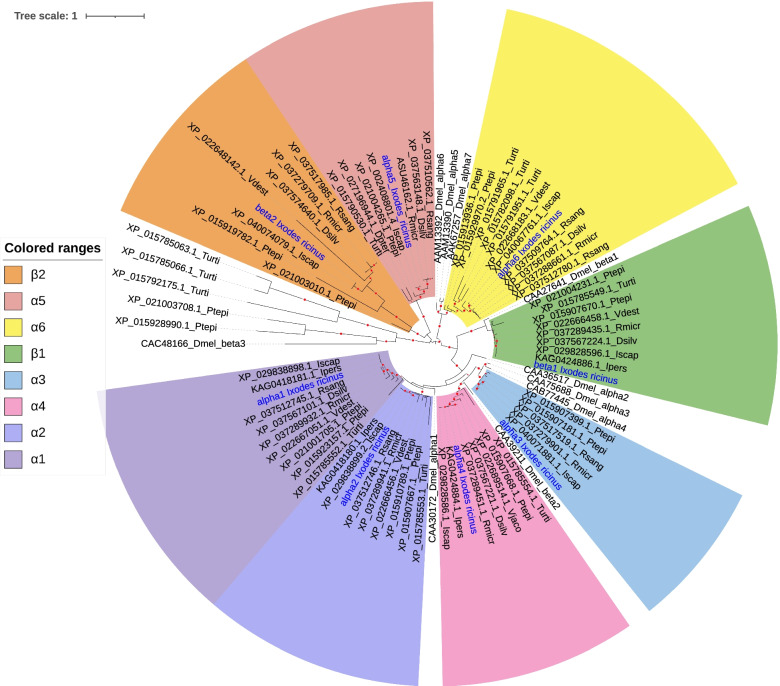
Maximum-likelihood phylogeny of nAchRs. The phylogeny includes eight sequences identified in the synganglion transcriptome of the tick *I. ricinus* and their homologs in Arachnida (other tick species, other Parasitiformes, Acariformes) or in *Drosophila melanogaster*. Labels indicate the accession numbers of protein sequences, and species name (abbreviated as Dmel for *D. melanogaster*, Ptepi for *Parasteatoda tepidariorum*, Vdest for *Varroa destructor*, Vjaco for *Varroa jacobsoni*, Turti for *Tetranychus urticae*, Dpter for *Dermatophagoides pteronyssinus*, Rmicr for *Rhipicephalus microplus*, Rsang for *Rhipicephalus sanguineus*, Dsilv for *Dermacentor silvarum*, Iscap for *Ixodes scapularis*, Ipers for *Ixodes persulcatus*). Labels for *I. ricinus* sequence are in blue. Filled circles on branches indicates bootstrap support (support increases with circle width, ranging from 80 to 100)

The ML phylogenetic study of tick nAChRs, including outgroups from other Arachnida and from an insect, *D. melanogaster,* allowed us to define eight clades with strong bootstrap support (Fig. 4). Because genes of this family have been named and numbered independently in each species, and due to duplication or gene loss in different branches, a consistent naming system valid for all species is impossible, so we numbered clades based on orthogroups in Arachnida. Four clades named α1–4 were related to four genes in *D. melanogaster* (Dmelα1, Dmelα2, Dmelα3 and Dmelβ2), but there was no one-to-one orthology between the genes from Arachnida and from *D. melanogaster* respectively. For example the α1 and α2 clade were co-orthologous to Dmelα1 whereas the α3 clade of Arachnida was orthologous to the pair of co-orthologs Dmelα2 and Dmelα3. The α6 and β1 clades were orthologous to *D. melanogaster* co-orthologs Dmelα5–6-7 and Dmelβ1, respectively. A second beta-type clade of Arachnida genes, named β2, was present both in ticks and other Arachnida but had no homologs in the genome of the house-fly nor in other insect genomes. This was also the case of a clade of alpha-type copies, that we named α5. Last, we identified a group of divergent nAChR genes (characterized by very long branches) comprising both the sequence Dmelβ3 and sequences found in the spider *Parasteatoda tepidariorum*, but apparently without orthologs in ticks.

### Phylogeny of the non-AChR cysloop-LGICs

**Fig. 5:**
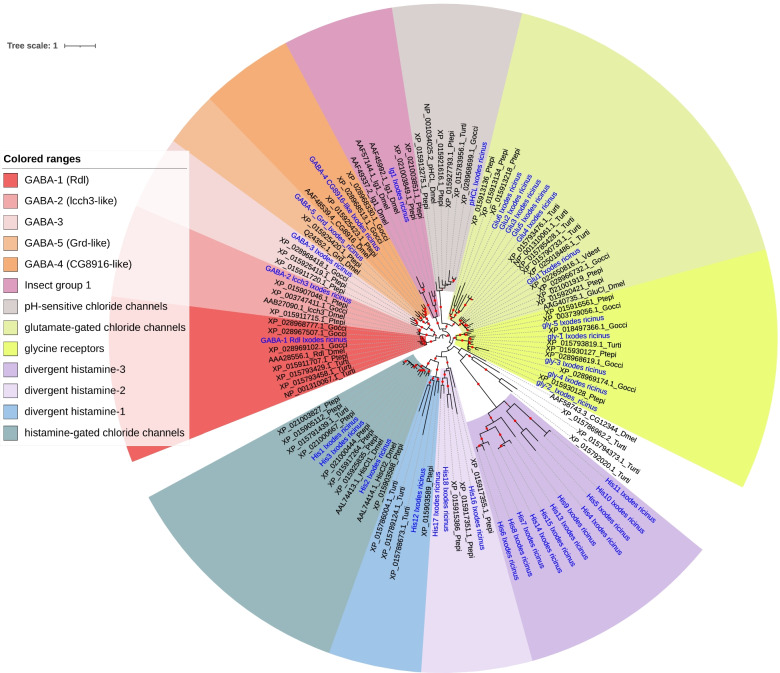
Maximum-likelihood phylogenetic tree of cys-loop LGICs, excluding nAChRs. The phylogeny includes sequences from the tick *I. ricinus* and representative arthropods: *P. tepidariorum* (house spider), Acariformes, Parasitiformes, and *D. melanogaster*. Labels indicate accession numbers of protein sequences and abbreviated species name (abbreviations as in Fig. 4, plus Gocci for *Galendromus occidentalis*). Labels for *I. ricinus* sequence are in blue. Filled circles on branches indicates bootstrap support (support increases with circle width, ranging from 80 to 100)

The numbers of different cysLGICs in *I. ricinus* and other representative arthropods are summarised in Table 3: the total was 21 in the house-fly, while 54 genes were found in the house spider (*P. tepidariorum*) and 46 in *I. ricinus.* Based on our phylogenetic tree supported by high bootstrap values, we could define eight major clades of non-AChR cysloop-LGICs in Arachnida, comprising ticks and predatory mites (Parasitiformes, e.g. *Galendromus occidentalis*), plant-feeding mites (Acariformes, e.g. *Tetranychus urticae*) and spiders (Fig. 5):

**Table 3:**
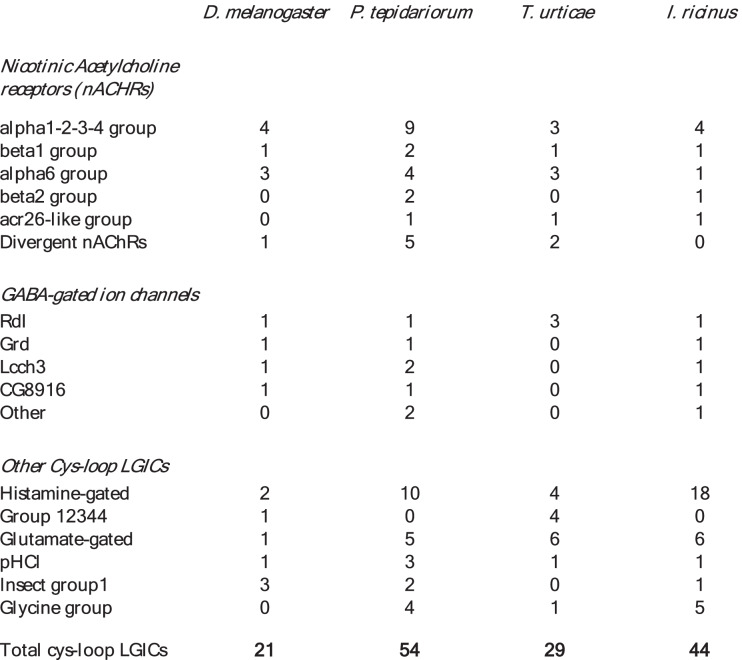
Numbers of cys-loop LGICs in *D. melanogaster* and in three Chelicerata, the house spider *P. tepidariorum*, the mite spider *T. urticae* and the tick *I. ricinus*

i) GABA-group: five different sub-groups were found, including GABA-1, corresponding to the “Resistant to dieldrin” (Rdl) homologs, GABA-2, corresponding to “ligand-gated chloride ion channel homolog 3” (lcch3), GABA-3 which was represented in several Arachnida groups, but not in *D. melanogaster*, GABA-4, corresponding to homologs of the CG8916 gene in *D. melanogaster*, and GABA-5, representing “GABA and glycine-like receptor” (grd) homologs. Of note, Rdl in *I. ricinus* was characterized by three alternative isoforms: their sequences were identical except in a stretch of 154 amino acids, which did align well but showed several substitutions, suggesting the presence of alternative exons. We analysed the genomic data of *I. scapularis* and identified a triplication of an exon, which explains the presence of these alternative transcripts (Fig. S1).
ii) pH-sensitive chloride channels (pHCl): a single homolog was identified for *I. ricinus*, compared to three in *P. tepidariorum*.
iii) “insect group 1”: this clade comprised three copies in *D. melanogaster*, with one homolog in *I. ricinus* and two in *P. tepidariorum*.
iv) Glutamate gated chloride channels: within this group, we could distinguish one “conserved” subclade - one homolog in *I. ricinus* (Glu1) but multiple copies in *P. tepidariorum* (2) or *T. urticae* (5) - and one “divergent” subclade represented in *I. ricinus* (5 copies) and *P. tepidariorum* (3 copies).
v) Glycine receptors were represented by multiple copies in several groups of Arachnida, with 5 copies in *I. ricinus*.
vi) Histamine-gated chloride-channels (HisCl): three copies in *I. ricinus* (His1, His2 and His3) were found to be homologous of the genes HisCl1 and HisCl2 in *D. melanogaster*.
vii) other clades: we found three more clades represented only in Arachnida, or only in ticks, all characterized by long branches, i.e. by divergent sequences. This was the case of a clade with one copy in *I. ricinus* (His 12): this clade grouped robustly with the Histamine clade, so we named it “Divergent histamine 1”. A second clade, related to these genes, but with lower bootstrap support (< 80) comprised genes in *I. ricinus* (His16–17-18), and also three genes in *P. tepidariorum*: we named it “Divergent histamine 2”. The third clade, characterized by particularly divergent sequences, comprised as many as 14 copies in *I. ricinus* (named His4 to His15) for which we found no homolog in other Arachnida. In our phylogeny, this group branched with the gene CG12344 from *D. melanogaster*, but with low bootstrap support. Based on the genomic organization in *I. scapularis* (Fig. S2) we found that eight of these copies (His4-His11) formed a cluster of tandem copies immediately adjacent to genes of the conserved HisCl clade (*I. scapularis* sequences homologous to His1, His 2 and His3). This very close physical proximity suggest that all of these copies probably emerged through tandem duplication, and that the digergent clade (His4 to His 11 and His 13 to His15) and His-gated subunits have a common ancestor. We then named it “Divergent histamine 3”.

### Evolution of cysLGICs in ticks

For a finer-scale study of each sub-group of the cysLGICs, we also made phylogenies including homologs from other tick species (Fig. S3). For GABA, «Insect group 1», and phCl, we found a pattern of one-to-one homology between ticks and other organisms, with no genic expansion in ticks. For GABA-1 (Rdl), however, some of the tick species are represented by two different sequences, but we found for both *I. ricinus* and *I. scapularis* that they correspond to alternative transcripts (Fig. S1). Two sequences were also present for respectively the ticks *Dermatocentor silvarum* and *Rhipicephalus microplus*, differing at the same characteristic sites (for example the Ala^301^ to Ser mutation at the second position of the M2 segment of RDL). We also note that *Galendromus occidentalis* (Acari: Parasitiformes), an outgroup of ticks, also retain the same polymorphism among three copies of GABA-1. For GluCls, Glycine and Histamine receptors, we found genic expansions in all tick species compared to other organisms. This was particularly the case in the His group, with multiple duplications in the different tick species, whereas long branches in the expanded sub-clades indicate an accelerated evolution of these sequences.

### Functional expression of Rdl in *Xenopus laevis* oocytes

A full-length cDNA was obtained by RT-PCR, using the primers designed using the *Rdl* contig. The corresponding cRNA was then micro-injected into *Xenopus laevis* oocytes in order to monitor the potential functionnal expression of the RDL receptor. When challenged with 100 μM GABA,

the micro-injected *Xenopus* oocytes responded with robust currents in the μA range (Fig. S4A), providing functional evidence of the expression of RDL. To further characterize this effect, the GABA concentration–response relationship was obtained by applying increasing concentrations of GABA (range 1–300 μM). The GABA concentration-response curve revealed an EC_50_ value of 19.95 μM (log EC_50_ = − 4.7 ± 0.035, *n* = 18), with current amplitudes normalized to the maximal response obtained to 300 μM GABA (Fig. S4B).

## Discussion

### A meta-transcriptome of high completeness, enriched by synganglion transcriptome sequencing

Our work is based on the high throughput sequencing of a synganglion transcriptome, a first for the tick *Ixodes ricinus*, but also on the combination of these data with two previous assemblies of tick whole body transcriptomes. This allowed us to obtain a meta-transcriptome of very high completeness (> 96% BUSCO completeness), an essential step for comparing expression levels in the synganglion and in the whole body. This approach eliminated a large part of the noise associated with de novo RNA-Seq assemblies (our two assembled synganglion transcriptomes each had > 600 K contigs), as we obtained a compact reference assembly with ~ 70 K contigs only. This reduction, due to the filtering of contigs with very low read support, could be associated with a loss of information, but high completeness metrics of the meta-transcriptome indicate that this risk was reduced. Overall, it appears that our newly acquired transcriptomic data for the synganglion has greatly improved the level of completeness of the meta-transcriptome, thanks to the inclusion of many transcripts that are highly specific to the synganglion.

### High expression specificity and neuronal signature of the synganglion

The DE study comparing synganglion and whole body samples showed a strong association between genes up-regulated in the synganglion and neuronal/neurotransmission functions. A potential bias, especially given that we used ticks sampled from the wild, could be the presence of pathogens or parasites which might interact with the synganglion and alter expression patterns [3]. As patterns of contamination might have been unequal among libraries, this could have biased our comparisons. However, the strong clustering of libraries by tissue and condition suggests that this factor has not played a major role in expression patterns. A second possible limitation of our approach is that the tissues we dissected and determined to be the synganglion could have been contaminated by adjacent tissues or cells, for example due to the particular organisation of this tissue which is crossed by the oesophagus and surrounded by the fat body. Although it is difficult to rule out this possibility entirely, the high specificity and distinct functional profile of the synganglion libraries indicate that contamination must have been limited. Enriched categories in the synganglion include neuropeptides, ion channels, etc., many of which were either completely absent or represented by partial sequences in transcriptomes obtained from the whole body or other tissues. A large majority of cysloop-LGICs were also upregulated in the synganglion, which is expected for these ion channels known to be expressed in neurons, which are particularly concentrated in the CNS. This overexpression concerns even divergent sequences of the cysLGICs (in the GluCls, or His-gated groups expanded clades), suggesting that these subunits are active mainly in the tick CNS. For the His-gated group, however, we note the exception of His12 (“divergent Histamine-1” clade) which was downregulated in the synganglion. A few of the cysLGIC genes were not differentially expressed (Synganglion vs Whole body comparison), including the homolog of ‘Insect group 1’ (a group comprising CG7589, CG6927, and CG11340 in *D. melanogaster*). This is consistent with a study that showed that IG1 is non-neuronal in this species [27].

Although our study focused primarily on one class of ion channels, the cys-loop LGICs, our assemblies (the synganglion assemblies as the meta-transcriptome) provide rich material for gene discovery in many functional groups associated with neurotransmission or neuronal functions in general. For example, we found that several of the genes with the highest log-fold variation in expression in the synganglion have vertebrates homologues which have been extensively studied for their association with Alzheimer’s Disease: this is the case for the APP-like peptide (APPL) and of tau protein [28], as well as a homolog of TIA-1, a gene associated with the development of tau protein [29]. Interestingly, a putative tau homolog was also identified in the synganglion transcriptome of another tick species, *Dermacentor variabilis*, where it has been reported to be strongly up-regulated upon initiation of blood feeding (unfed versus partially-fed ticks) [8].

In addition to this, in our study, a contig identified as Cathepsin B was among the most strongly downregulated genes in the synganglion. Cathepsin B has been shown to have complex interactions with Alzheimer’s Disease and to be involved in the degradation or production of Abeta, as reviewed in [30]. In *D. melanogaster*, APPL has been shown to be restricted to the nervous system, and the authors hypothesized that a neural-specific function encoded by the APP gene has been selectively maintained along evolution [31]. In the tick synganglion, the strong up-regulation (tau, APP, TIA1) or down-regulation (CathB) illustrates the interest of arthropod models to study the expression profiles of these genes, their interactions and their evolution.

### Profiles of downregulated genes in the synganglion

For a complete understanding of tick neurobiology, it is also interesting to assess which genes are downregulated in the synganglion. Genes in this category showed enrichment in terms associated with iron binding, heme binding, and aromatase activity, in particular. In a previous study [26] where we investigated expression in the whole body of the tick, comparing fed and unfed conditions, genes downregulated during feeding (i.e. significantly more expressed in the whole body of unfed ticks than of fed ticks) were particularly enriched in the same first three terms. We found that these terms were co-occurring in the same genes, most of which of the cytochrome P450 (cytP450) family. This suggests that one group of cytP450 tends to be particularly expressed in the whole body of unfed ticks, but is expressed at a much lower level both in the whole body of partially fed ticks and in the synganglion of ticks of all conditions.

### Differentiation between the “fed” and “unfed” state

Our design allowed to compare the expression levels in the synganglion of respectively unfed ticks (males or females) and partially fed female ticks. The transcriptomes of these two conditions were comparatively less differentiated than the synganglion and whole body samples (11 and 36% of DE genes in the two comparisons, respectively). Although the lower percentage of DE genes could be in part explained by a question of statistical power (only eight samples were used in this comparison, three for fed ticks and five for unfed ticks), the study of all samples still indicates a lower influence of the feeding status than of the tissue (synganglion or whole body). Up-regulated genes upon feeding (fed+ genes) were enriched in chitin-associated proteins, a result that appears surprising given the absence of cuticle in this tissue. In a previous study of expression in the whole-body [26], cuticle associated proteins were also the most up-regulated genes upon feeding, suggesting that an increased expression of cuticle-related products upon feeding is pattern shared between different tissues, including the synganglion. To determine the intersection of changes upon feeding (in the synganglion) and of genes of expression among tissues (synganglion vs whole body), we counted the genes that were both differentially expressed in the fed vs unfed comparison and up-regulated in the synganglion: with this filtering, up-regulated genes in the fed condition were down to 79 (from 2243) whereas down-regulated genes were down to 1362 (from 2305). This suggests that extremely few genes up-regulated in the synganglion were Fed+, and then that the ‘cuticle’ signature of Fed+ genes is not specific to the synganglion, but is a global pattern in the tick body. By contrast, a relatively large fraction of genes were at the same time Syn+ and down-regulated in the synganglion upon feeding. Consistently, Fed- genes had functional profiles relatively similar to Syn+ genes (which can be seen comparing Table 2 and Supplementary Table 4). Overall, this indicates that the expression profile of the synganglion of unfed ticks has a more distinct profile (compared to the whole body) than the synganglion of feeding ticks. We observed this precise pattern for most of the cys-loop LGIC gene (most of them being Syn+), since their expression levels were often comparatively lower in the fed samples than in the unfed samples, as was the case of GABA-1 (Rdl), Glu-6, PhCl for example. We propose that the synganglion overall activity could be lowered when ticks are fixed on a host and start to feed, due to the down-regulation of several functions associated with mobility or sensorial activities related to host-search, for example. This can be compared to another study of the synganglion of unfed, part-fed and replete ticks [8]: in this study, expressions profiles of the latter two stages were relatively similar, whereas the unfed state had the most dissimilar profile, and the “replete” stage showed a decline in expression and specificity of expression, which was linked to a possible shift from active stages of the tick life-cycle (host search, feeding, mating) to late stages (oviposition, senescence and death). Whereas our design allowed to compare only two time-points (respectively unfed and partially-fed ticks), previous RNA-Seq based studies point to a more complex picture where expression patterns change sequentially along the blood-meal [32, 33]. For unfed ticks, we also expect that the time since the last meal (i.e., the age of ticks) could also be an important factor. Future studies, where these factors would be controlled (age of unfed ticks, different phases of the blood-meal, presence/absence of pathogens) will be necessary to more fully apprehend metabolic changes in the tick CNS in the life-cycle of these organisms.

### Completeness of cys-loop LGICs

Our synganglion transcriptomes allowed us, thanks to a careful manual curation, to obtain a remarkably large and complete collection of cys-loop LGICs compared to previous studies, even when based on complete genome sequences: for example, a compilation of cys-LGICs in the *I. scapularis* genome [4] reported a total of 32 genes, less than the 44 found in our study. In the recent study of five tick complete genomes [13], no specific compilation of cys-LGICs was reported, but our phylogenetic studies and the alignments we obtained indicate that for all these species, the cysLGIC record is less complete than in our study, with several genes entirely missing or having only partial sequences. This difference may be explained by difficulties associated with assembling and annotating the complex genomes of ticks, which could be particularly an issue for genes with a neuronal pattern of expression, which tend to have an increased gene size both in vertebrates and invertebrates [34]. Our analysis of gene models and gene sizes in the genome of *I. scapularis* indeed indicates that some of cys-loop LGICs have very long gene sizes (e.g. the RDL gene spans > 247 Kb). Such long genes may represent a difficulty for prediction tools, or may span different contigs or scaffolds, resulting in incorrect or incomplete gene models. We hypothesize that this factor could explain the fact that sequences of the cysloop LGIC predicted from tick genomes are relatively incomplete, compared to our transcriptome based sequences. Overall, this illustrates the high value of high quality transcriptome data for the tick CNS.

### Tick-specific patterns of duplications for cysLGICs

The inclusion of other tick species in phylogenetic analyses of the different sub-groups allowed us to precise patterns of gene duplication within teh group of ticks. We found that ticks have a repertoire comparable to other arthropods for nAChRs and for GABA receptors, but show gene-family expansions in other categories of receptors (GluCls, Gly, and even more strikingly in the His family). In *I. scapularis* (based on its genome) and *I. ricinus* (based on its transcriptome) we found that an internal duplication (triplication of an exon) in the gene GABA-1 (RDL) allows the production of three alternative transcripts, either with or without a mutation confering resistance to cyclodienes. Interestingly, we found that several tick species from other genera and and also an outgroup species (*G. occidentalis*, Acari: Parasitiformes) possess the same polymorphism, which was also observed in *Varroa destructor* [35] and in insects (e.g. in *D. melanogaster* [36]). This polymorphism is therefore probably ancient in ticks, and cannot have occurred as the result of selective pressure caused by synthetic insecticides. We rather hypothesize that as in other groups [35, 37], it may represent a mechanism enhancing tolerance to naturally occuring compounds, probably of plant origin.

## Conclusions

Our new transcriptome data for the tick synganglion, allowed us to build a high quality reference transcriptome for *Ixodes ricinus*, strongly enriched in transcripts specific to or at least strongly over expressed in neurones. This allowed us reconstructing a large catalogue of the tick LGICs and uncovering tick-specific patterns of gene duplication for some groups of these receptors. Based on these synganglion transcriptomic ressources, we achieved the first functional reconstitution of a GABA-gated channel for *Ixodes ricinus*, made of the RDL subunit. Future studies will be needed to evaluate the pharmacological and biophysical properties of RDL and of other neuroreceptors, including sensitivity to insecticides.

## Methods

### Tick sampling

Ticks (*Ixodes ricinus*) were collected under two conditions, unfed or partially fed. Unfed adult female and male ticks were collected by the flagging method on vegetation in Carquefou (Loire-Atlantique, France). Partially fed adult females (estimated before the phase of rapid engorgement, i.e. at ~ 5–6 days of attachment [38]) were collected in Chizé (Deux-Sèvres, France), from roe deers captured in the framework of a long-term monitoring programme of these mammals in the Chizé Forest, a biosphere Reserve. The permission to collect ticks from roe deers of the Forest of Chizé was obtained from the relevant forest governer body, the Office National de la Chasse et de la Faune Sauvage (ONCFS) and approved by the Director of Food, Agriculture and Forest (Prefectoral order 2009–14 from Paris). All methods were carried out in accordance with relevant guidelines and regulations.

### Sample preparation

Ticks were dissected under a stereomicroscope within 2–3 days of collection. Whole ticks were washed in RNase-ExitusPlus™ plus water. Synganglia were excised, rinsed in PBS buffer 1X, and dried by placing them gently on a clean sheet of paper. Synganglia were then placed in RNase-free microfuge tubes containing RNA extraction buffer (RA1 buffer, Nucleospin RNA XS kit, Macherey Nagel) and stored at − 80 °C until use. For RNA extraction, the tubes were gently thawed and tissues were mechanically disrupted with decontaminated pestles (3%H2O2) and eluted in 200 μl of RA1. Samples were prepared for three conditions, unfed females, males, and partially fed females, each with three replicates (Table 4). Samples were made with pools of synganglia (*n* = 25 for unfed females or males, *n* = 10 for partially fed females). RNA was extracted using the Nucleospin RNA XS kit (Macherey Nagel, Düren, Germany), adapted for small samples, with a 5 U DNAse treatment.

**Table 4:**
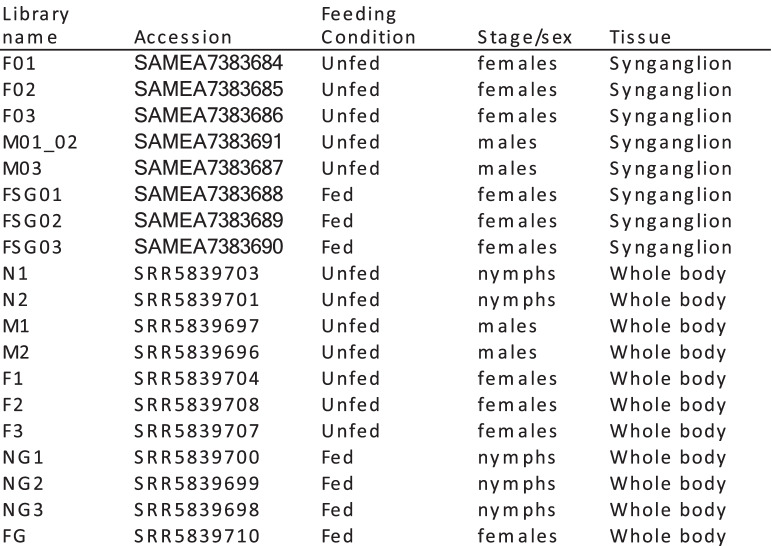
List of libraries used for the comparison of expression levels in the synganglion and in the whole body. Accession: Accession numbers of reads (synganglion libraries, generated for the present study, were deposited as BioProject PRJEB40724, whereas whole body samples correspond to BioProject PRJNA395009, produced in a previous work of our group [26]). Feeding condition: ticks were either unfed (collected on the vegetation) or fed (semi-engorged state of repletion, ticks collected on roe deers for the synganglion libraries and on mice or rabbits for the whole body libraries). Stage/sex: indicates if ticks were female or male (for adults), or nymphs (the sex of nymphs could not be determined). Tissue: libraries were obtained from the dissected synganglion (this study) or from tick whole bodies (already published sequence data)

### RNA dosage and quality

RNA purity was assessed with a NanoDrop (Thermo Fisher Scientific Inc., Watham, MA, USA). The commonly used threshold to consider samples as sufficiently pure is A280/A260 > 1.80. Three out of nine samples were just below that threshold (F03, M01 and M02) but were kept for further analyses. Quantity was assessed with a Qubit (Invitrogen, Thermo Fisher Scientific Inc., Watham, MA, USA): quantities were low in the three samples mentioned above (they were not even dosable), but ranged between 68 and 304 ng for other samples. Finally, a further assessment of quantity and quality was conducted with the 2100BioAnalyzer instrument (Agilent Technologies Inc., Santa Clara, CA, USA): quality was determined by peak profiles, which was good for all samples, with a single, clear peak corresponding to rRNA, but low quantities for three samples, in particular M01 and M02. We decided to pool the latter two samples to make a single library, named M01_02.

### Library preparation and sequencing

RNA-Seq library preparations were carried out from 60 ng total RNA, or the total volume when not quantifiable (for F03, M01_02). We used the NEBNext Ultra II directional RNA library prep kit (NEB, Ipswich, MA, USA), which allows mRNA strand orientation (sequence reads occur in the same orientation as anti-sense RNA). Briefly, poly(A) + RNA was selected with oligo (dT) beads, chemically fragmented and converted into single-stranded cDNA using random hexamer priming. Then, the second strand was generated to create double-stranded cDNA. cDNA were then 3′-adenylated, and Illumina adapters were added. Ligation products were PCR-amplified. Ready-to-sequence Illumina libraries were then quantified by qPCR using the KAPA Library Quantification Kit for Illumina Libraries (KapaBiosystems, Wilmington, MA, USA), and libraries profiles evaluated with an Agilent 2100 Bioanalyzer (Agilent Technologies, Santa Clara, CA, USA). Each library was sequenced using 151 bp paired end reads chemistry on a Illumina HiSeq 4000 sequencer, producing a minimum of 40 million paired-reads for each library.

### Read cleaning and assembly

Short Illumina reads were bioinformatically post-processed as in Alberti et al. 2017 [39] to filter out low quality data. First, low-quality nucleotides (Q < 20) were discarded from both read ends. Then remaining Illumina sequencing adapters and primer sequences were removed and only reads ≥30 nucleotides were retained. These filtering steps were done using in-house-designed software based on the FastX package (https://www.genoscope.cns.fr/fastxtend). Finally, read pairs mapping to the phage phiX genome were identified and discarded using SOAP aligner [40] (with default parameters) and the Enterobacteria phage PhiX174 reference sequence (GenBank: NC_001422.1). In addition, ribosomal RNA-like reads were detected using SortMeRNA [41] and filtered out. This set of cleaned reads were deposited to EBI under the BioProject accession PRJEB40724. The quality of cleaned reads was evaluated with fastQC, using MultiQC for visualization [42], all parameters being satisfactory. Potential duplicates were removed with the the tool fastuniq (https://sourceforge.net/p/fastuniq/). The assembly was done with Trinity (version 2.3.2) [43] with the options normalize-reads and RF (to account for strandedness). Calculations were done on the Genotoul Bioinformatics platform, using 100GB of memory. We made two separate assemblies, for respectively unfed ticks (females and males) and feeding ticks (females only). This choice was motivated by the expectation of different profiles of transcription in both conditions [44] and of a reduced risk of producing chimeric contigs compared to a single assembly of all the reads. Potential presence of pathogens or parasites (bacteria, viruses, eukaryotic parasites), or contamination by other organisms, was assessed using Kraken 2 [45], a *k*-mer based taxonomic assignation tool.

### Construction of a high quality reference transcriptome

The two assemblies produced for the synganglion transcriptomes were combined with two previously published transcriptomes of the tick whole body - assembly accessions GFVZ [26] and GIDG [46] in the TSA division of Genbank - to produce a reference meta-transcriptome (list of libraries in Table 4). The rationale for mixing different sources of transcribed sequences was to obtain the most complete transcriptome possible, in order to better evaluate the specificity of transcripts in the synganglion versus all tissues combined (the whole body). We did not use other transcriptomes published for specific organs of *I. ricinus*, because they have been sequenced a relatively low coverage and show comparatively low completeness [26]. To obtain the meta-transcriptome, we used the DRAP software (RunMeta) [47], a dedicated tool able to fuse contigs of different assemblies in a unique representative contig set. This tool allows to reduce redundancy within and between sets, and to eliminate low quality contigs with low read support. Completeness of the different transcriptome assemblies was assessed with BUSCO (version 4.0.2) [48], using the arachnida_odb10 set of *n* = 2934 conserved genes.

### Transcriptome annotation

Annotation of the reference transcriptome was done following the Trinotate pipeline (https://github.com/Trinotate/Trinotate.github.io) [49]. First, genes were predicted with TransDecoder. Predict (https://github.com/TransDecoder/TransDecoder/wiki), a prediction guided by PFAM-A searches with hmmscan (to recognize conserved domains) and a homology search with Diamond [50] blastp (https://github.com/bbuchfink/diamond) between long peptides and the uniprot_sprot database. Predicted peptides were then searched with SignalP (to determine the presence of signal peptides), tmhmm (to determine if transmembrane domains are present) and rnammer (to identify rRNA contigs). We also performed homology searches against the UniRef90 database, with Diamond blastp (for predicted peptides) and Diamond blastx (for transcripts). Finally an annotation report was generated to combine all results, including retrieved Gene Ontology terms, KEGG [51], Eggnog and Pfam domains.

### Annotation of cys-loop LGIC genes in the reference transcriptome

A collection of reference cys-loop LGIC proteins comprising genes from *D. melanogaster* and the mite *T. urticae* [14] (Acari, Acariformes) was established, to be used as tblastn queries on the reference meta-transcriptome of *I. ricinus*. A careful examination of the matches was then performed, to remove any remaining redundancy. Based on similarities within contigs and preliminary phylogenetic analyses, we identified events of chimerism among different gene sequences, which occured in the GABA and nAChR families respectively. For these genes, we extracted matching reads, which were re-assembled with CAP3 [52], using stringent parameters. This allowed to disentangle chimerisms and reconstructing complete sequences within gene families. Once we achieved the annotation of cys-loop LGICs and occasional correction of the inital contigs, these genes were re-integrated in the meta-transcriptome: to avoid redundancy, the edited and curated sequences were used and substituted to the corresponding initial contigs, and were annotated with the same Trinotate pipeline.

### Read counts and differential expression analyses

Alignment of reads with the meta-transcriptome including the curated cys-loop LGIC sequences were performed with Bowtie2(v2.4.1)-RSEM(v1.3.1), using default parameters. Expression levels were measured using the “count per million” metric produced by RSEM. Levels of expression were measured for eight libraries corresponding to the synganglion (produced for this study), but also, for comparison, for part of the “whole body” libraries used to build the reference meta-transcriptome as described above. Note that the «whole body» libraries correspond to all tissues of adult ticks, including the synganglion. Given the small size of this tissue, we expect however that transcripts from the synganglion constitute a small fraction of all transcripts, and therefore that the «whole body» represents an acceptable proxy for a control in the comparison between the synganglion and other tissues combined. Libraries from the GIDG transcriptome were not used because they were not replicated by condition. Read counts from the BioProject PRJNA395009 (GFVZ TSA assembly) were used, for all libraries except four shown to be of lesser quality in a previous study: library names and their accession are given in Table 4. First, to determine which transcripts were more expressed in the synganglion, we made a differential expression analysis (DE) comparing synganglion and whole body libraries, with edgeR and limma [53, 54]. Second, to compare the patterns of expression in the synganglion of unfed and partially fed ticks (hereafter “unfed” and “fed” conditions), we used the read counts from the synganglion libraries only. The same methods were used in both DE analyses. We used the function gene_to_trans_map of edgeR, which assigned read counts to the same gene for contigs defined as isoforms based on their Trinity name (i.e. contigs that differed only by the final _i suffix). This resulted in 63,194 unique genes. The statistics of each gene (read count, log-fold -change, significance, and annotation) were visually explored with the Glimma package [55]. Criteria for defining an up- or down-regulated gene were an absolute log-fold change > 2 and an adjusted *P*-value (adj-*P*-value) < 0.05). The topGO package was used for the analysis of GO enrichment in genes up- or down-regulated in the synganglion (the whole body being taken as the reference) and for the analysis of enrichment in genes up- or down-regulated in the synganglion-“fed” libraries (the “synganglion-unfed” condition being taken as the reference) - (Alexa A, Rahnenfuhrer J (2021). *topGO: Enrichment Analysis for Gene Ontology*. R package version 2.44.0). Lists of differentially expressed genes for the two comparisons (synganglion versus whole body, and synganglion of fed ticks versus synganglion of unfed ticks) with their annotation and read counts are given in Table S5.

### Functional expression of tick neuroreceptors

The coding sequence of *Rdl* was PCR-amplified using pecific primers designed based on the synganglion transcriptome assembly (Iri-RDL-F0 GGTCAAGGAGGTCGCTTGCC; Iri-RDL-R0 ACGACAACTTTAAAGGCGAATGC, FuIri-RDL-ptb-XhoF AGCGATGGCGTTCAGTTGCTG; FuIri-RDL-ptb-ApaR GACCGTGTGCACTATTCGTCG). The *Rdl* PCR-product was subcloned into the transcription vector pTB-207 [56] using the In-Fusion® HD Cloning Kit (Clontech™) and cRNAs were synthesized with the T7 mMessage mMachine kit (Ambion™). Expression of GABA-Rdl in *Xenopus laevis* oocytes and two-electrode voltage clamp electrophysiology were carried out as described previously [57]. Briefly, 36 nL of 700 ng/μL *Rdl* cRNAs were micro-injected into defolliculated *Xenopus* oocytes (Ecocyte Bioscience) using a Drummond Nanoject II microinjector and incubated 3 days at 19 °C to allow subunit expression. Electrophysiological recordings were performed using an Oocyte Clamp OC-725C amplifier (Warner Instruments) under voltage clamp at − 60 mV and analyzed using the pCLAMP 10.4 package (Molecular Devices).

### Phylogenetic analyses of the cys-loop LGICs

Because nAChRs constitute a divergent group within cysLGICs, we analysed separately nAChRs and all other cysLGICs. We included in these phylogenetic analyses sequences of *I.ricinus* (from our annotation) and of other tick species, plus homologs of other Acari and of *D. melanogaster* Alignments were made with MUSCLE, cleaned with GBLOCKs and further edited manually to preserve conserved blocks. Phylogenetic maximum likelihood trees were obtained with IQ-TREE [58] the best model of substitution being determined with Model Finder [59], and branch support being assessed with 1000 ultrafast bootstrap replication [60]. Phylogenetic trees were formatted graphically with ITOL [61].

## Supplementary Information

**Additional file 1: Table S1.** Taxonomic assignation of the reads with Kraken 2. First column, taxonomic categories or taxa. Next columns, for each synganglion library (label in the first line), percentage of the reads assigned to the corresponding taxon.

**Additional file 2: Table S2.** Top 20 enriched Gene Ontology terms among genes significantly down-regulated in the synganglion. Columns, GO IDs and terms for Molecular Function, Biological Process and Cellular localization; Annotated : number of transcripts associated with each GO; Significant: number of genes annotated with this term in the enriched category. Expected: expected number of genes. Weight01: level of significance of the enrichment.

**Additional file 3: Table S3.** Top 20 enriched Gene Ontology terms among genes significantly up-regulated in the “fed” conditions. Comparison between the “fed” and “unfed” condition, for synganglion libraries only.

**Additional file 4: Table S4.** Top 20 enriched Gene Ontology terms among genes significantly down-regulated in the “fed” condition. Comparison between the “fed” and “unfed” condition, for synganglion libraries only.

**Additional file 5: Table S5.** Lists of differentially expressed genes with their annotation and read counts for all libraries. Columns: Gene ID in the meta-transcriptome, Best BLASTX hit against the SwissProt database, Best BLASTX hit on theUniref90 database, list of GO terms associated, normalized reads counts in Fragments per Kilobase per Million reads (FPKM) for each libraries (Synganglion or Whole Body, Fed or Unfed ticks, as precised by the column title. Below each each library name, the terms «Up», «Down» or «NI» respectively indicate up-regulation, down-regulation, or that the library was not included in the statistical analysis. Sheet A (A – Syn+ genes): comparison between the synganglion and whole body libraries, *n*=8483 genes up-regulated in the synganglion. Sheet B (B – Syn- genes): comparison between the synganglion and whole-body libraries, *n*=5040 genes down-regulated in the synganglion. Sheet C (C – Fed+ genes, Syn libraries only): comparison between the synganglion of fed ticks and the synganglion of unfed ticks, genes up-regulated in fed ticks. Sheet D (D – Fed- genes , Syn libraries only): comparison between the synganglion of fed ticks and the synganglion of unfed ticks, genes down-regulated in fed ticks.

**Additional file 6: Table S6.** GenBank accessions of the cysloop LGICs, First column, short name of each gene as used in the meta-transcriptome. All sequences were reconstructed in this study and deposited by our group, based on new synganglion assemblies, except gly-1 and gly-2 for which we used contigs sequences already published in GenBank.

**Additional file 7: Figure S1.** Genomic structure of the gene GABA-1-Rdl, with three predicted isoforms and their relative expression. A: Reannotation of the gene XP_042145571.1 located on a genomic scaffold of *Ixodes scapularis*, NW_024609839.1. The gene (drawing not to scale) has a span of ~247 kbp, on a scaffold of ~92 Mbp. The whole gene is on the minus frame. Boxes correspond to exons. Grey-filled boxes correspond to exons which we consider incorrect (over-predictions in both cases, based on the conserved sequence of GABA-1-Rdl) and by comparison with the homologous sequence in other tick species. Exons in blue correspond to a predicted triplication of one exon (exon 7a, 7b, 7c), whereas only one exon (7b) was annotated for XP_042145571.1. For each numbered exon, the positions indicate the start and end. Numbers followed by a star correspond to reannotations and differ from the published sequence. The translated sequence of each exon is given. B: Alignment of the translated sequences for the three alternative exons 7, including both the sequences for *I. scapularis* based on our reannotation and the homologous sequences from the *I. ricinus* alternative transcripts identified in the synganglion transcriptome (this study). Two trans-membrane domaines are indicated, and an arrow shows the A->S mutations known to confer resistance to dieldrin. C: Relative expression (y-axis) of the three isoforms of GABA-1-Rdl. Expressions was counted as counts per millions with RSEM, and normalized to evaluate relative expression. In x-axis, different synganglion libraries produced in this study (described in Table 4). In blue, red and yellow, estimated relative expression of exon 7a, 7b and 7c respectively.

**Additional file 8: Figure S2.** Genomic organization of Histamine-gated-like sequences in *Ixodes scapularis.* We used the Histamine gated-like sequences obtained from our meta-transcriptome of *Ixodes ricinus* to search homologous genes in *I. scapularis* (homology inferred from near-identity of protein sequences), and to locate them on the genome. The figure shows the entire scaffold NW_024609883 (109.621.045 bp) from the *I. scapularis* genome, which has homologs to His1 to His12 in *I. ricinus*. The upper scale indicates positions in Mbp. The lower scale represents a focus on a smaller region containing clusters of His-like sequences, with a scale in Kbp. Annotated genes and their orientations are indicated by filled triangles, with below, the accession of the protein sequence in *I. scapularis*, and the name of its homologous sequence in *I. ricinus* (this study). A star indicates that the gene model is probably incorrect: XP_042142979 matches with His1 only over the first four exons, while its remaining sequence appears to represent a chimeric fusion with a totally different gene, XP_040070355 is missing an N-term, XP_040070356 is incomplete and matches only the beginning of His3, whereas XP_040068404 matches only the end of His3 and is in opposite frame of XP_ 040070356 (we interpret this as a likely error of the genome assembly, the two accession probably representing respectively the start and end of the same gene). Open triangles indicate regions where no gene has been annotated in *I. scapularis*, but where we detected high similarities with *I. ricinus* genes (for His4 and His9 respectively). For His13 to His18, homologous regions were detected on different scaffolds.

**Additional file 9: Figure S3.** Maximum-likelihood phylogenetic trees of different sub-groups of cys-loop LGICs. Phylogenies includes sequences from different ticks species (labels and branches in blue) and other arthropods: *P. tepidariorum* (house spider), Acariformes, Parasitiformes, and *D. melanogaster*. Labels indicate accession numbers of protein sequences and species name. Accessions of *I. ricinus* are listed in Table S6. Filled circles on branches indicate bootstrap support (support increases with circle width, ranging from 80 to 100). Trees were rooted based on the complete phylogeny (Fig. 5). A: GABA group, B: «Insect group 1», C: pHCL, D: GluCls, E: Gly, F: His.

**Additional file 10: Figure S4.** Concentration–response relationship of GABA on the *I. ricinus* RDL receptor expressed in *Xenopus* oocytes. A. Representative current traces of a single oocyte micro-injected with *Iri-rdl* cRNA perfused with increasing concentrations of GABA for 10 seconds (short bars). The concentration of GABA (μM) is indicated above each trace. B. Concentration–response curve for GABA on the Iri-RDL channel. All current responses are normalized to 300 μM and shown as the mean ± SEM.

## Data Availability

Read sequences of the synganglion transcriptomes have been published to EBI as BioProject PRJEB40724. The nucleotide sequences of cysLGICs have been deposited to GenBank as MZ027281-MZ027288 (*n* = 8 nAChRs) and MZ099581-MZ099616 (other cysLGICs, *n* = 36 transcripts, *n* = 34 different genes) as detailed in Table S6. In addition, the gly1 and gly2 sequences correspond to accessions GIDG01020391.1 (gly1, coding sequence 1–1978) and GIDG01020392.1 (gly2, coding sequence 1–1993). Contig sequences of the meta-transcriptome, their annotation table, interactive plots of the Synganglion-vs-Whole body differential expression analysis, non-aligned and aligned protein sequences of the cysLGICs are available in a public repository (https://data.inrae.fr): Rispe, Claude, 2021, “Transcriptome of the the tick synganglion”, 10.15454/FGTIHR“.

## Abbreviations

nAChRs: Nicotinic acetylcholine receptors
CNS: Central Nervous System
cys-loop LGICs: Cys-loop ligand-gated ion channels
cysLGICs: Cys-loop ligand-gated ion channels
MD plot: Mean_difference plot
MDS plot: Multidimensional scaling plot
