# Correction: Transcriptome of the synganglion in the tick *Ixodes ricinus* and evolution of the cys-loop ligand-gated ion channel family in ticks

**DOI:** 10.1186/s12864-022-08740-0

**Published:** 2022-07-12

**Authors:** Claude Rispe, Caroline Hervet, Nathalie de la Cotte, Romain Daveu, Karine Labadie, Benjamin Noel, Jean-Marc Aury, Steeve Thany, Emiliane Taillebois, Alison Cartereau, Anaïs Le Mauf, Claude L. Charvet, Clément Auger, Elise Courtot, Cédric Neveu, Olivier Plantard

**Affiliations:** 1 INRAE, Oniris, BIOEPAR, Nantes, France; 2 Department of Biology and Biotechnology “L. Spallanzani”, University of Pavia, Pavia, Italy; 3 Génomique Métabolique, Genoscope, Institut de biologie François Jacob, CEA, CNRS, Université d’Evry, Université Paris-Saclay, Evry, France; 4 Université d’Orléans, LBLGC USC INRAE 1328, 1 rue de Chartres, 45067 Orléans, France; 5 INRAE, ISP, 37380 Nouzilly, France

**Correction: BMC Genomics 23, 463 (2022)**

**https://doi.org/10.1186/s12864-022-08669-4**

Following publication of the original article [1], it was reported that Fig. 3 was mistakenly published as a duplicate of Table 2. The correct Fig. 3 is provided in this Correction article and the original article [1] has been updated.

**Fig. 3:**
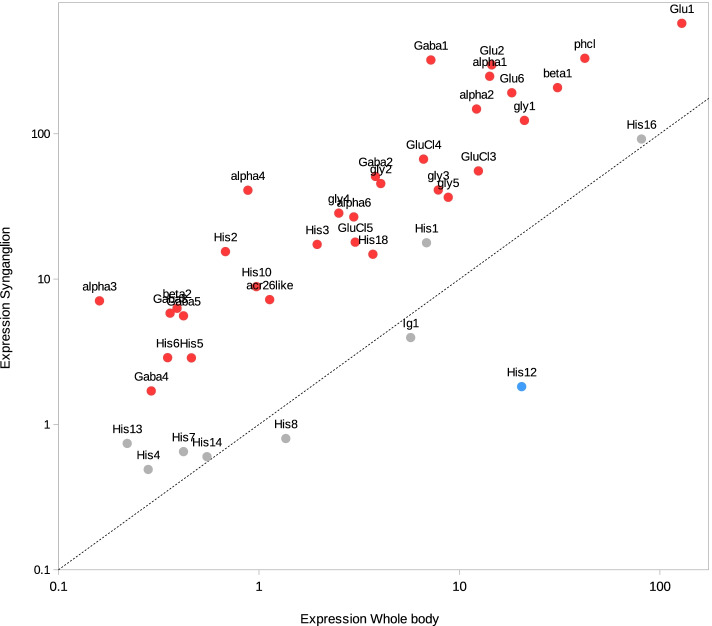
Comparison of expression counts for synganglion and whole body, for the cys-loop LCICs. Expression was measured in counts per million reads (cpm) with RSEM. Expression counts were averaged for all synganglion libraries (y-axis) and for all whole body libraries (x-axis). Colors of dots indicate if genes were found to be significantly up-regulated in the synganglion (red), down-regulated in the synganglion (blue) or with no sig. Difference (grey). The dotted-line corresponds to y = x. For both axes, a logarithmic scale was used. Corresponding accessions are listed in Table S6
