# Supplementary figures and images for: Transcriptome of the synganglion in the tick Ixodes ricinus and evolution of the cys-loop ligand-gated ion channel family in ticks
Source: BMC Genomics. 2022 Jun 23;23:463. doi: 10.1186/s12864-022-08669-4 (PMC9219234; doi:10.1186/s12864-022-08669-4)

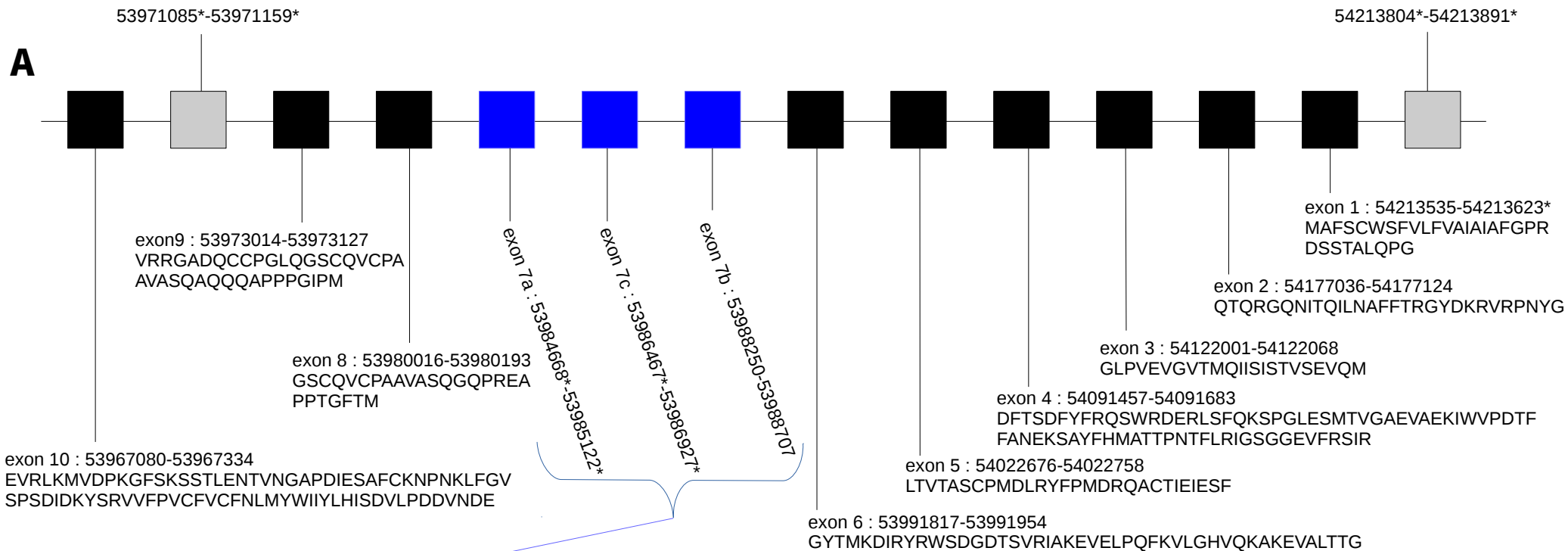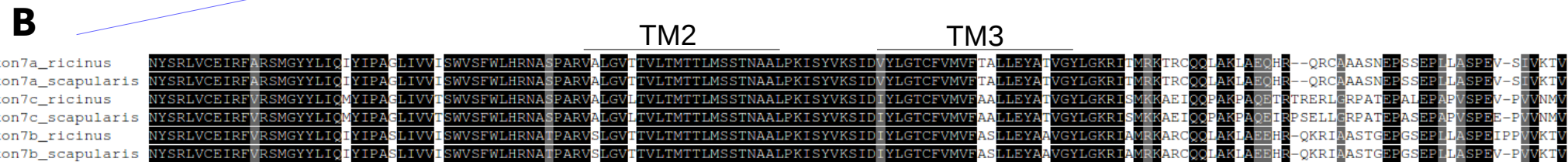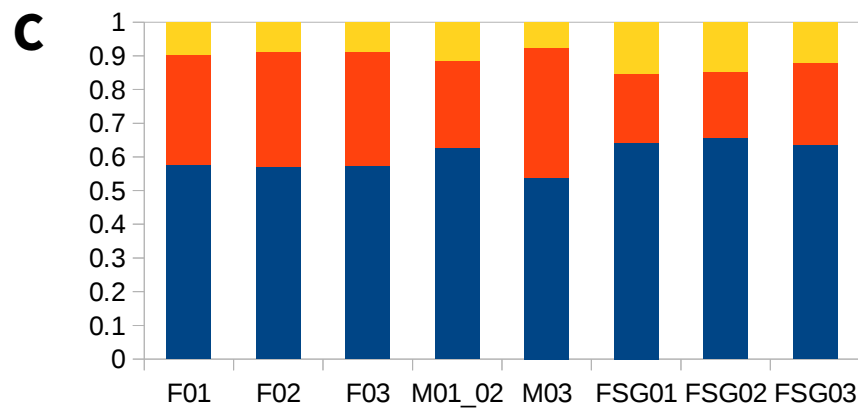

Supplement: Supplementary file 7 — Additional file 7: Figure S1. Genomic structure of the gene GABA-1-Rdl, with three predicted isoforms and their relative expression. A: Reannotation of the gene XP_042145571.1 located on a genomic scaffold of Ixodes scapularis, NW_024609839.1. The gene (drawing not to scale) has a span of ~247 kbp, on a scaffold of ~92 Mbp. The whole gene is on the minus frame. Boxes correspond to exons. Grey-filled boxes correspond to exons which we consider incorrect (over-predictions in both cases, based on the conserved sequence of GABA-1-Rdl) and by comparison with the homologous sequence in other tick species. Exons in blue correspond to a predicted triplication of one exon (exon 7a, 7b, 7c), whereas only one exon (7b) was annotated for XP_042145571.1. For each numbered exon, the positions indicate the start and end. Numbers followed by a star correspond to reannotations and differ from the published sequence. The translated sequence of each exon is given. B: Alignment of the translated sequences for the three alternative exons 7, including both the sequences for I. scapularis based on our reannotation and the homologous sequences from the I. ricinus alternative transcripts identified in the synganglion transcriptome (this study). Two trans-membrane domaines are indicated, and an arrow shows the A->S mutations known to confer resistance to dieldrin. C: Relative expression (y-axis) of the three isoforms of GABA-1-Rdl. Expressions was counted as counts per millions with RSEM, and normalized to evaluate relative expression. In x-axis, different synganglion libraries produced in this study (described in Table 4). In blue, red and yellow, estimated relative expression of exon 7a, 7b and 7c respectively. [file 12864_2022_8669_MOESM7_ESM.pdf]

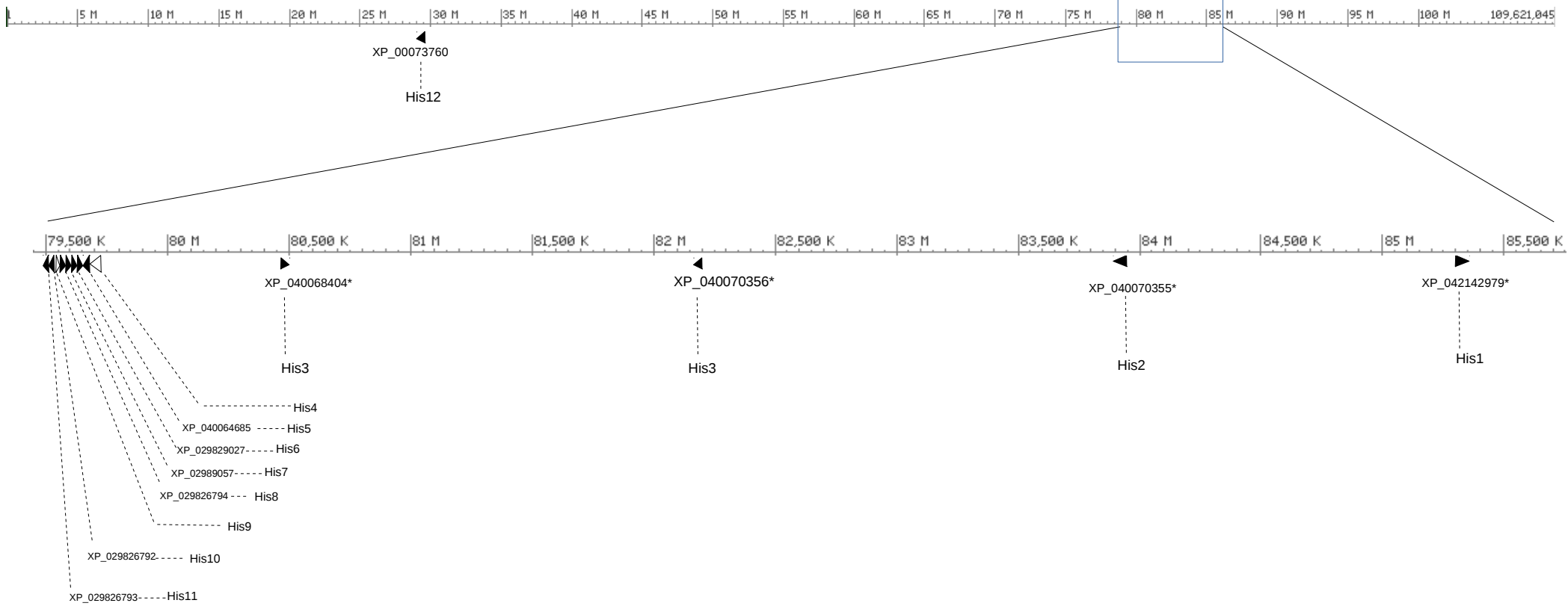

Supplement: Supplementary file 8 — Additional file 8: Figure S2. Genomic organization of Histamine-gated-like sequences in Ixodes scapularis. We used the Histamine gated-like sequences obtained from our meta-transcriptome of Ixodes ricinus to search homologous genes in I. scapularis (homology inferred from near-identity of protein sequences), and to locate them on the genome. The figure shows the entire scaffold NW_024609883 (109.621.045 bp) from the I. scapularis genome, which has homologs to His1 to His12 in I. ricinus. The upper scale indicates positions in Mbp. The lower scale represents a focus on a smaller region containing clusters of His-like sequences, with a scale in Kbp. Annotated genes and their orientations are indicated by filled triangles, with below, the accession of the protein sequence in I. scapularis, and the name of its homologous sequence in I. ricinus (this study). A star indicates that the gene model is probably incorrect: XP_042142979 matches with His1 only over the first four exons, while its remaining sequence appears to represent a chimeric fusion with a totally different gene, XP_040070355 is missing an N-term, XP_040070356 is incomplete and matches only the beginning of His3, whereas XP_040068404 matches only the end of His3 and is in opposite frame of XP_ 040070356 (we interpret this as a likely error of the genome assembly, the two accession probably representing respectively the start and end of the same gene). Open triangles indicate regions where no gene has been annotated in I. scapularis, but where we detected high similarities with I. ricinus genes (for His4 and His9 respectively). For His13 to His18, homologous regions were detected on different scaffolds. [file 12864_2022_8669_MOESM8_ESM.pdf]

Tree scale: 0.1

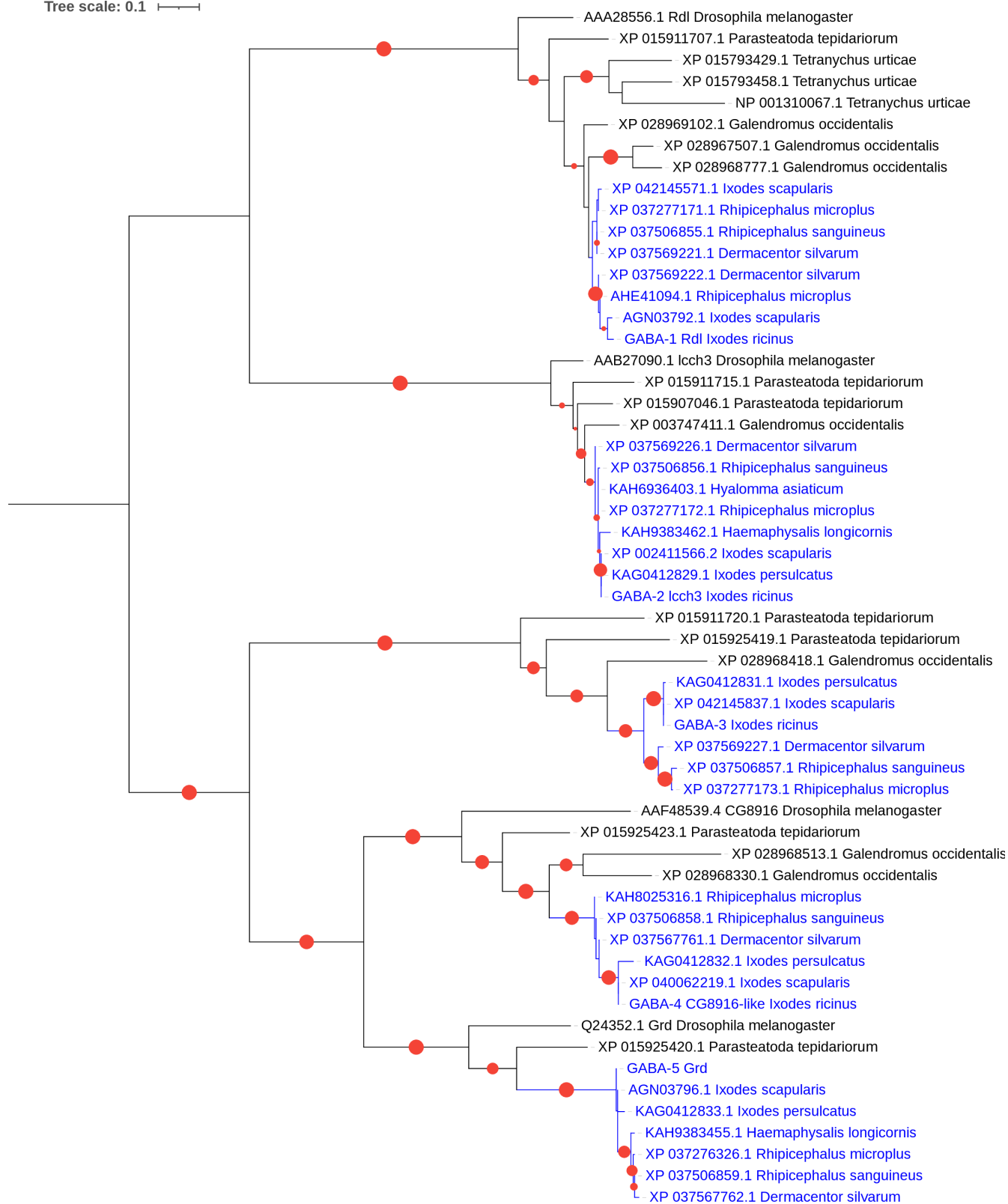

A

Tree scale: 1

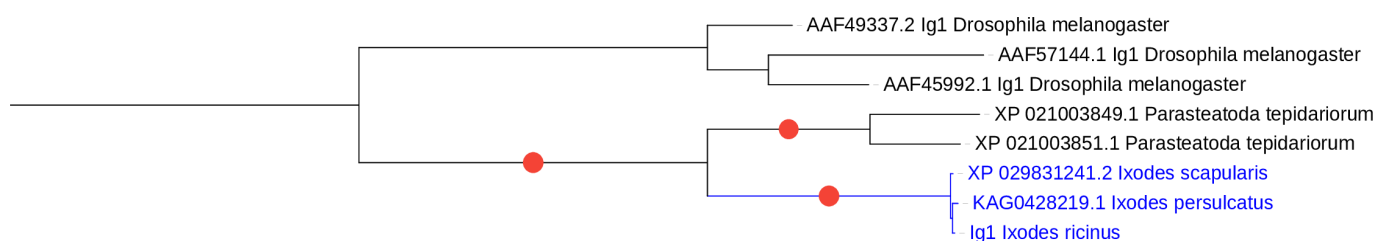

**B**

Tree scale: 0.1

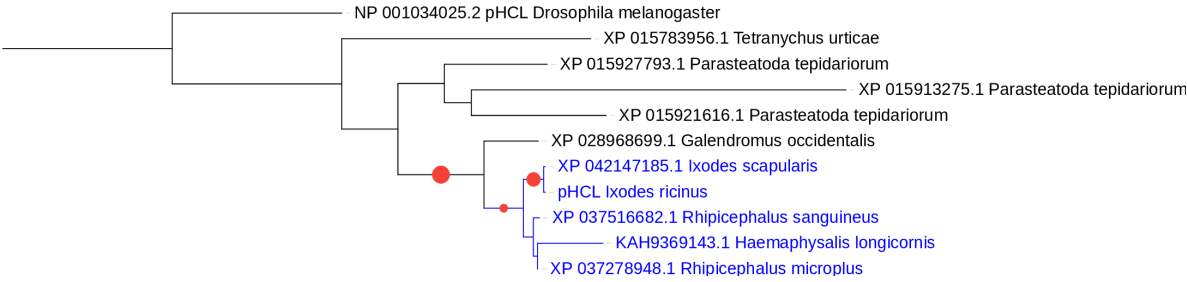

C

Tree scale: 1

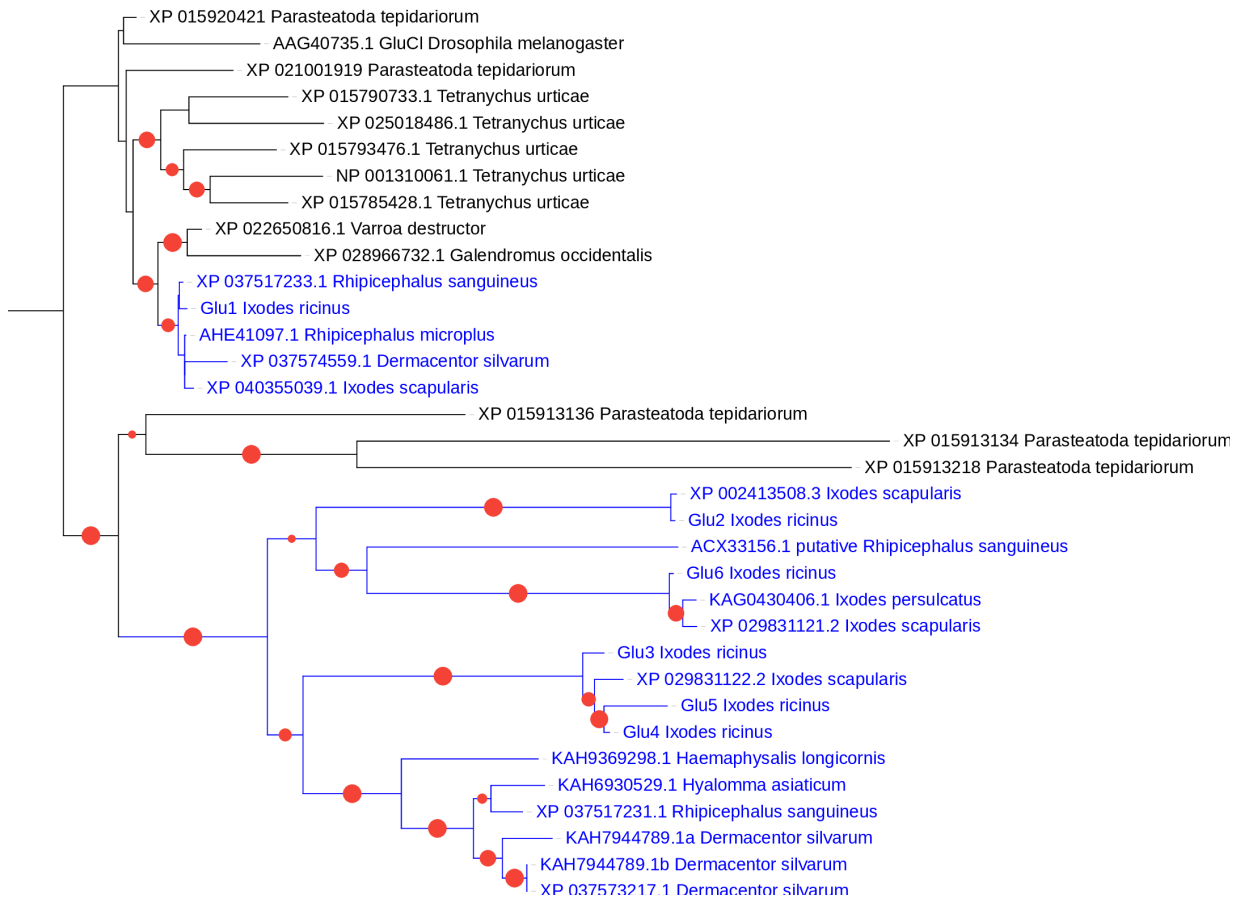

D

Tree scale: 1

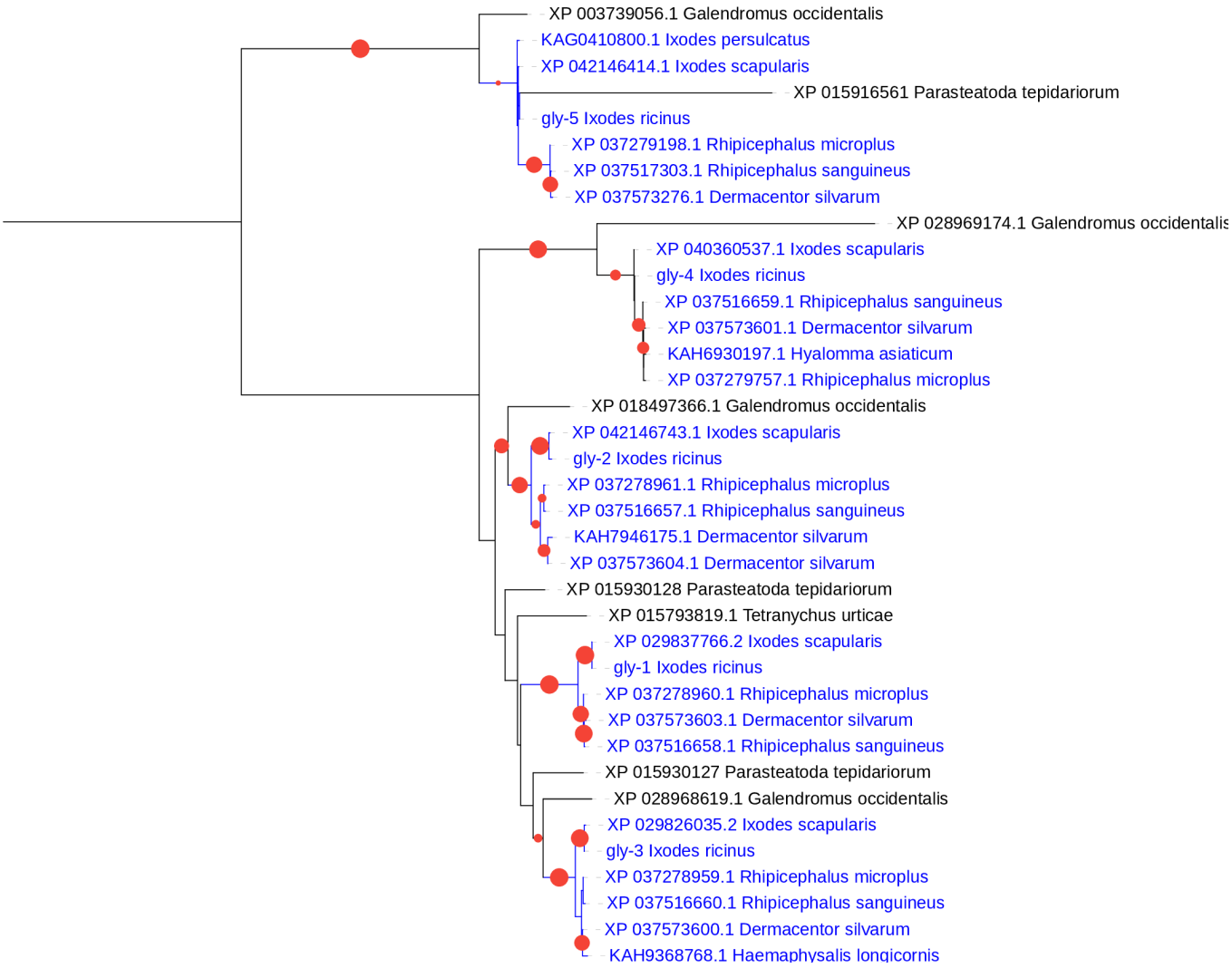

Tree scale: 1

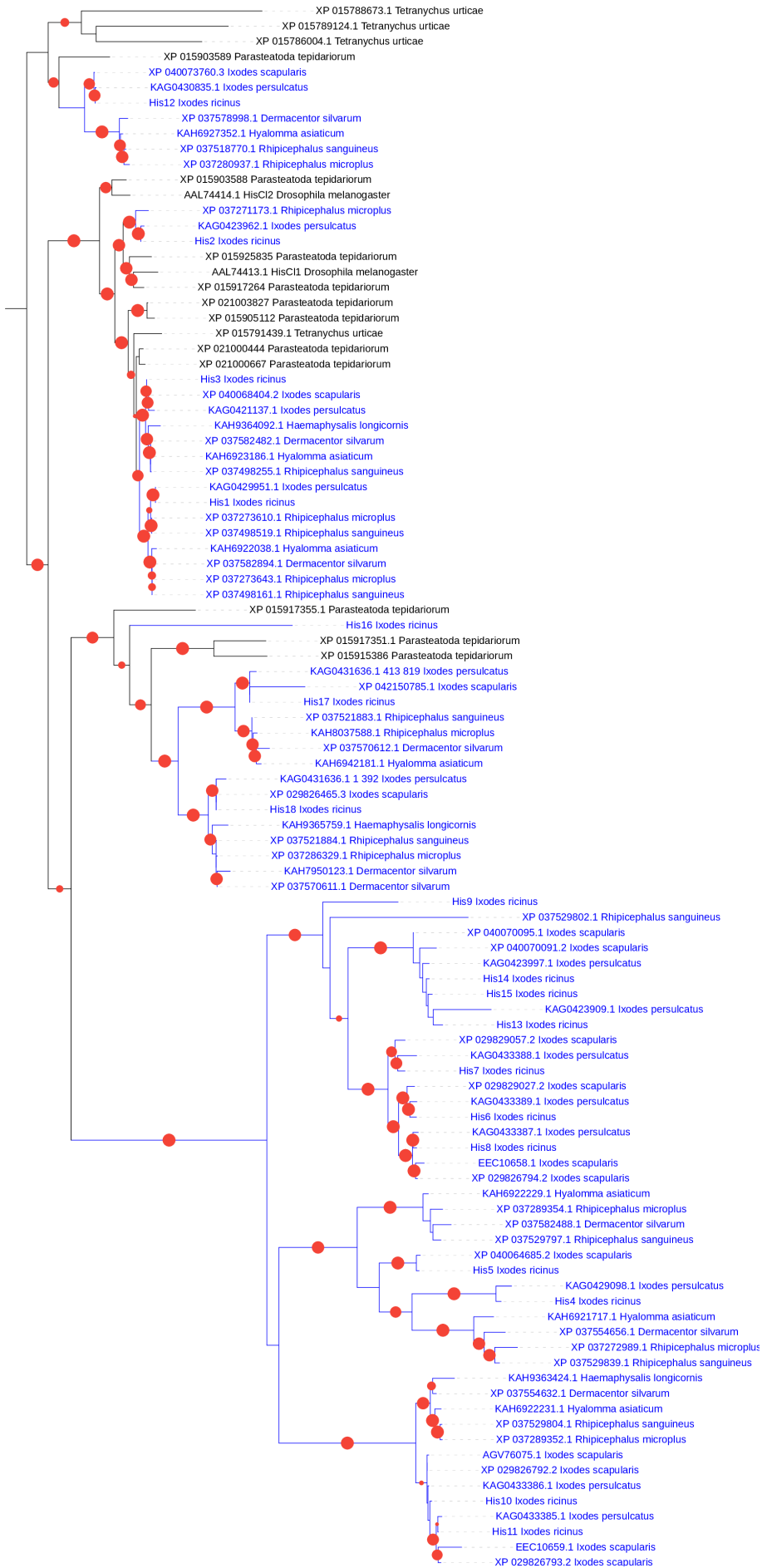

Supplement: Supplementary file 9 — Additional file 9: Figure S3. Maximum-likelihood phylogenetic trees of different sub-groups of cys-loop LGICs. Phylogenies includes sequences from different ticks species (labels and branches in blue) and other arthropods: P. tepidariorum (house spider), Acariformes, Parasitiformes, and D. melanogaster. Labels indicate accession numbers of protein sequences and species name. Accessions of I. ricinus are listed in Table S6. Filled circles on branches indicate bootstrap support (support increases with circle width, ranging from 80 to 100). Trees were rooted based on the complete phylogeny (Fig. 5). A: GABA group, B: «Insect group 1», C: pHCL, D: GluCls, E: Gly, F: His. [file 12864_2022_8669_MOESM9_ESM.pdf]

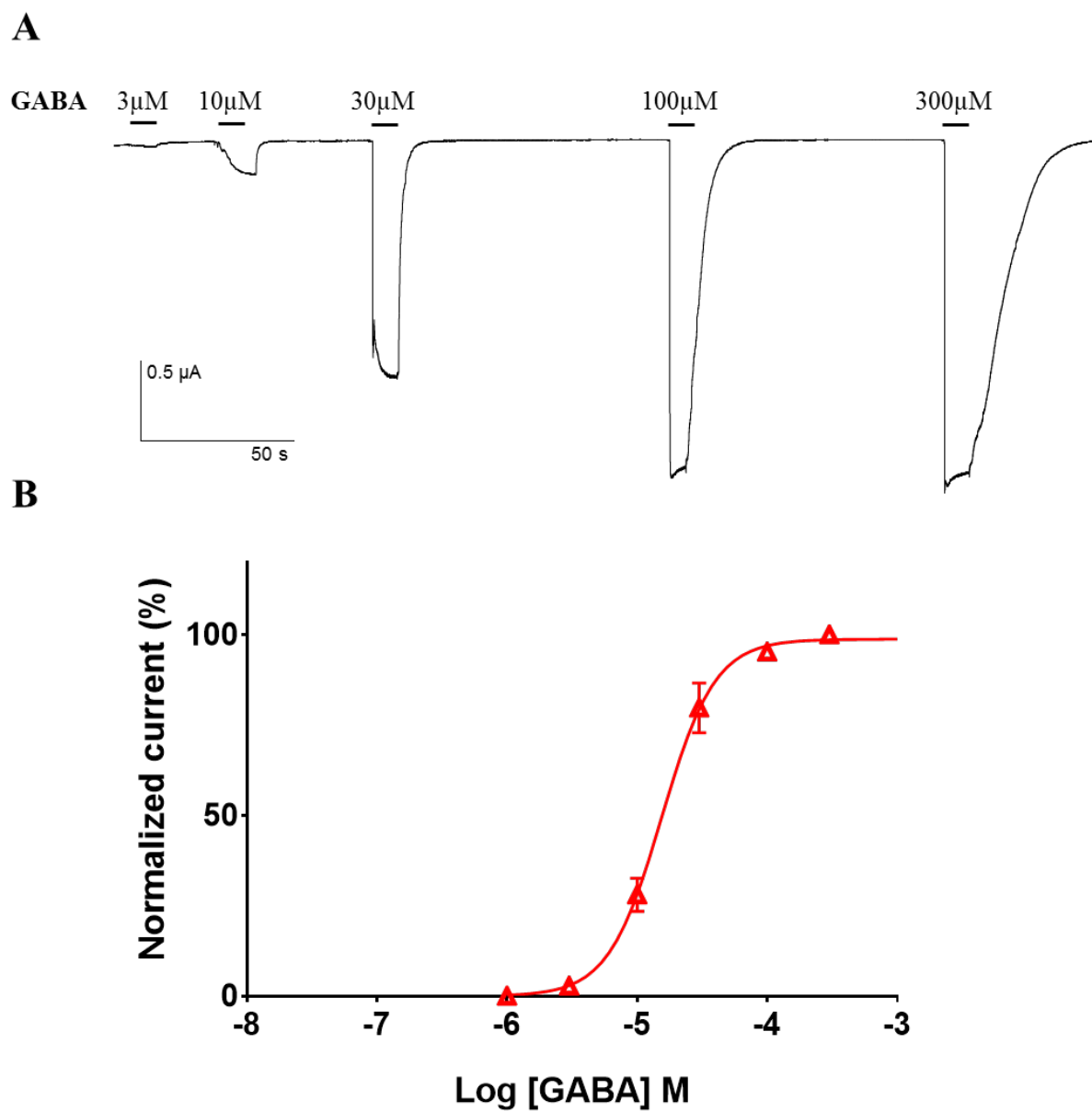

Fig. S4

Supplement: Supplementary file 10 — Additional file 10: Figure S4. Concentration–response relationship of GABA on the I. ricinus RDL receptor expressed in Xenopus oocytes. A. Representative current traces of a single oocyte micro-injected with Iri-rdl cRNA perfused with increasing concentrations of GABA for 10 seconds (short bars). The concentration of GABA (μM) is indicated above each trace. B. Concentration–response curve for GABA on the Iri-RDL channel. All current responses are normalized to 300 μM and shown as the mean ± SEM. [file 12864_2022_8669_MOESM10_ESM.pdf]
